# Characterizing local-scale heterogeneity of malaria risk: a case study in Bunkpurugu-Yunyoo district in northern Ghana

**DOI:** 10.1186/s12936-019-2703-4

**Published:** 2019-03-15

**Authors:** Punam Amratia, Paul Psychas, Benjamin Abuaku, Collins Ahorlu, Justin Millar, Samuel Oppong, Kwadwo Koram, Denis Valle

**Affiliations:** 1 School of Forest Resources and Conservation, University of Florida, Gainesville, USA; 2 Emerging Pathogens Institute, University of Florida, Gainesville, USA; 3 Noguchi Memorial Institute for Medical Research, University of Ghana, Legon, Accra, Ghana; 4 National Malaria Control Programme, Accra, Ghana

**Keywords:** Malaria, Bayesian, Fine-scale, Geostatistical, Ghana

## Abstract

**Background:**

Bayesian methods have been used to generate country-level and global maps of malaria prevalence. With increasing availability of detailed malaria surveillance data, these methodologies can also be used to identify fine-scale heterogeneity of malaria parasitaemia for operational prevention and control of malaria.

**Methods:**

In this article, a Bayesian geostatistical model was applied to six malaria parasitaemia surveys conducted during rainy and dry seasons between November 2010 and 2013 to characterize the micro-scale spatial heterogeneity of malaria risk in northern Ghana.

**Results:**

The geostatistical model showed substantial spatial heterogeneity, with malaria parasite prevalence varying between 19 and 90%, and revealing a northeast to southwest gradient of predicted risk. The spatial distribution of prevalence was heavily influenced by two modest urban centres, with a substantially lower prevalence in urban centres compared to rural areas. Although strong seasonal variations were observed, spatial malaria prevalence patterns did not change substantially from year to year. Furthermore, independent surveillance data suggested that the model had a relatively good predictive performance when extrapolated to a neighbouring district.

**Conclusions:**

This high variability in malaria prevalence is striking, given that this small area (approximately 30 km × 40 km) was purportedly homogeneous based on country-level spatial analysis, suggesting that fine-scale parasitaemia data might be critical to guide district-level programmatic efforts to prevent and control malaria. Extrapolations results suggest that fine-scale parasitaemia data can be useful for spatial predictions in neighbouring unsampled districts and does not have to be collected every year to aid district-level operations, helping to alleviate concerns regarding the cost of fine-scale data collection.

**Electronic supplementary material:**

The online version of this article (10.1186/s12936-019-2703-4) contains supplementary material, which is available to authorized users.

## Background

Over the past two decades, Ghana has made significant progress towards reducing malaria mortality [1]. This progress can be attributed to increasing coverage and improving access to rapid diagnostic tests and artemisinin-based combination therapy, implementing universal access to insecticide-treated bed nets, scaling-up indoor residual spraying (IRS) [2, 3] as well as climate change, urbanization patterns and infrastructural development [4, 5]. Despite these country-wide efforts for malaria control and prevention [6], and improved infrastructure, malaria morbidity remains relatively high [7]. The national early childhood (6 to 59 months old) malaria prevalence rate in Ghana has remained relatively stable (22–27%) from 2011 to 2016 [8]. However, it is important to consider that nationwide prevalence estimates mask significant spatial variability, as many parts of Ghana still experience intense seasonal malaria transmission, particularly in the northern regions [9, 10] where prevalence is greater than 40%. The 2016 Ghana Malaria Indicator Survey (MIS) revealed regional childhood prevalence estimates ranging between 5 and 31%, but these aggregated estimates may understate substantial heterogeneity within and between districts in each region [5].

Characterizing the spatial variation of disease prevalence by mapping exercises has proven to be useful for the strategic planning of malaria prevention and control activities at the national level [11–13]. In 2013, the Ghana National Malaria Control Programme (NMCP) collaborated with a team at Kenya Medical Research Institute-Wellcome Trust to develop a state-of-the-art map of malaria parasitaemia rates at the district level [5]. Their model estimates of *Plasmodium falciparum* in children aged 2 to 10 years in Northern Region ranged from 42% for the Tamale municipality to over 75% for most rural districts, presumably related to the residual malaria transmission in this area [14]. Linking data on climate and urbanization with locally available data yielded a more nuanced view of malaria distribution in the northern savannah compared to national averages, which allowed for the classification of districts into those that may be suitable/unsuitable for seasonal malaria chemoprophylaxis (SMC). Such work has paved the way for finer scale mapping efforts to describe the within-district local-scale heterogeneity in malaria parasite prevalence [15, 16], which is critical to increasing the effectiveness of current malaria control interventions implemented at the local level [17]. However, the ability to map at finer scale is contingent on the availability of rich surveillance data [18].

The purpose of this study was to characterize local spatial patterns in early childhood malaria prevalence in a single district in northern Ghana. A Bayesian hierarchical geostatistical model it was applied to a series of seasonal cross-sectional parasitaemia surveys conducted in the Bunkpurugu-Yunyoo district from 2010 to 2013, which were previously collected as part of an IRS intervention evaluation [3]. The high geographic resolution in these surveys provided a rich dataset for understanding the local scale malaria epidemiology in a residual transmission region [19]. Detailed surveys such as these can be prohibitively costly and would be untenable to scale to the entire Northern Region of Ghana, therefore exploratory work was conducted on how well the model could be extrapolated to unsampled neighbouring districts and how frequently these surveys would have to be implemented to achieve the same degree of resolution.

## Methods

The outcome used throughout this paper was microscopy-based malaria status of individuals sampled by parasitaemia household surveys that were conducted between October 2010 and March 2013 in the Bunkpurugu-Yunyoo district (BYD), Northern Region, Ghana. This dataset arises from a collaboration between the University of Ghana and the President’s Malaria Initiative (PMI) as part of an IRS evaluation project [20].

### Study area

Bunkpurugu-Yunyoo is located in Northern Region, Ghana (10.6° N 0.0° W). The district exhibits a gradual slope from the rocky Gambaga escarpment in the north/northwest to riverine plains in the south/southeast, dropping from 518 to 128 m above sea level within a relatively small area (30 km by 40 km). The district lies in the Guinea Savannah zone, characterized by a unimodal rainy season from May to October, which peaks in August–September while the remainder of the year is typically dry. The mean annual rainfall is between 100 mm and 115 mm, with annual temperature ranging between 30 °C and 40 °C. The predominant malaria vector species are *Anopheles gambiae* sensu stricto and *Anopheles funestus* [21]. In 2010, the estimated total district population was 122,591, with an estimated under-five population of 21,373. The study district is predominantly rural, with the exception of two settlements that exceed a population threshold of 5000, and are thereby designated as ‘urban’ according to the Ghana Statistical Service, namely Nakpanduri (population 6179) and Bunkpurugu (population 11,106) [22]. Most of the population live in mud-walled compounds and engage in rain-fed, small-scale farming as well as small-scale trading.

**Fig. 1:**
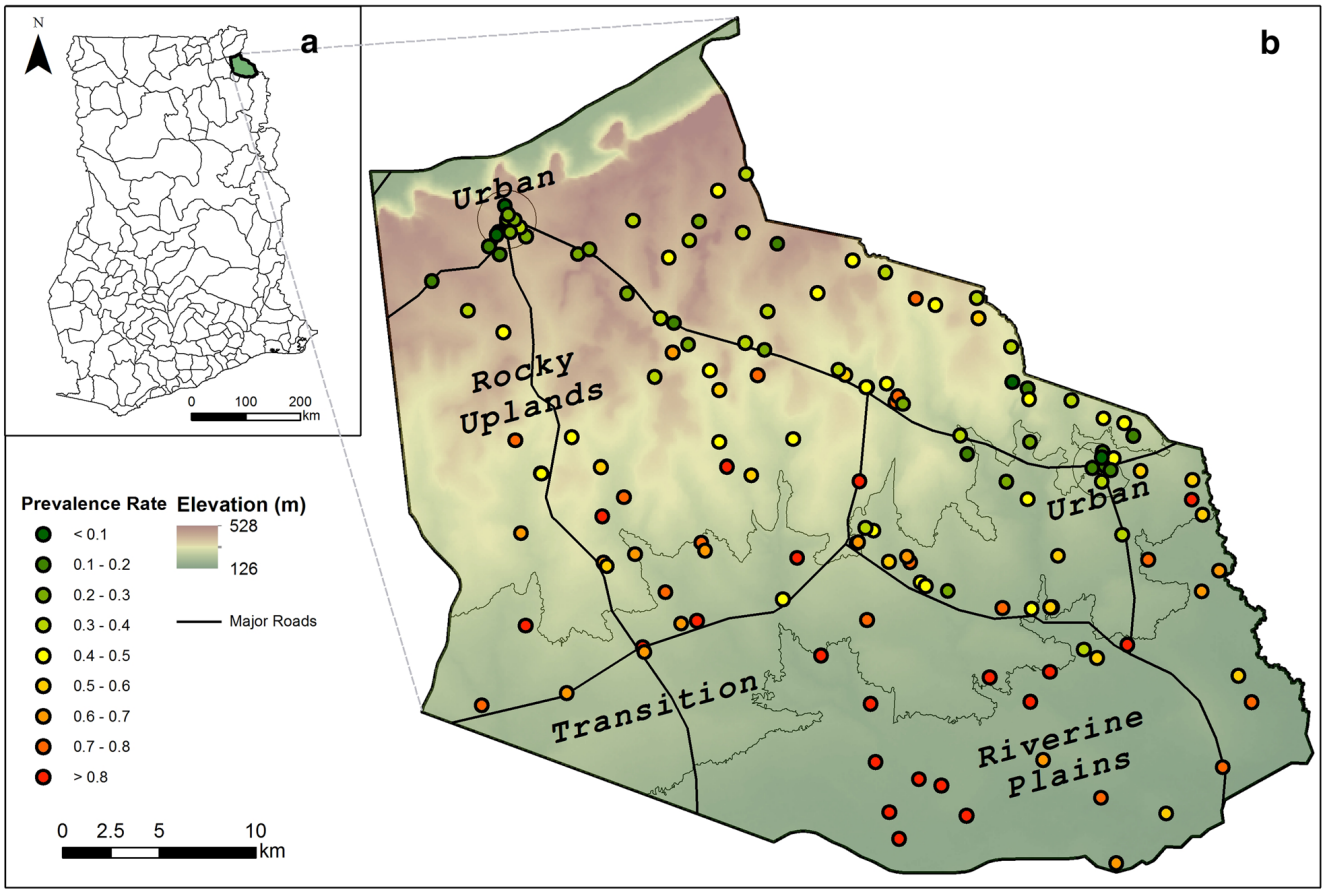
Communities sampled (black dots) in Bunkpurugu-Yunyoo district across all 2011–2013 surveys. The observed prevalence rates are displayed from low (green) to high (red) on the elevation surface and major roads. Four ecological zones defined by study design are highlighted by the elevation as rocky uplands, transition vegetation, riverine plains and urban zones. Inset map shows all the districts within Ghana, highlighting the Bunkpurugu-Yunyoo district

Malaria transmission in BYD is characterized by strong seasonal variations that closely follow rainfall patterns, with a peak lasting up to 3–4 months between August and November [6]. The study area is defined as the set of communities that were eligible for spraying by the PMI-funded IRS programme in 2011–2013 (Fig. 1). The study boundaries do not perfectly align with the official administrative district boundary because the sampling frame for the study consisted of the list of communities that fell under the public health jurisdiction of the BYD office of Ghana Health Service (GHS). As a result, this list leaves out communities handled by the East Mamprusi office of GHS along the extreme northeastern border and the southeastern corner of BYD.

The district had benefitted from two recent mass distributions of long-lasting insecticide-treated nets (LLINs). In 2010, the LLINs programme covered children under 5 years and pregnant women and in 2012 they covered all other members of the household, resulting in approximately > 75% coverage across BYD [2, 23]. The other major malaria prevention and control intervention in the region had been IRS, which began in 2011 using alphacypermethrin 0.4% WP (ICON^®^10CS, Syngenta, Basel, Switzerland) and shifted to organophosphates in 2013 due to declining susceptibility of *Anopheles spp.* to the pyrethroid insecticides [21].

### Malaria survey data

Surveys were conducted twice annually at periods of expected peak (i.e., the end of the rainy season, October–November) and trough (i.e., the end of the dry season, March–April) levels of malaria parasitaemia. Each survey was collected over a 2-week period.

Children under 60 months of age and their caregivers were randomly selected for each survey, using a multi-stage randomized cluster sampling design. Probability proportional to population was used to randomly sample approximately 72 communities per survey across the study area from a GHS roster of 238 communities. Within each community, survey teams visited 15–17 households with children under 5 years old, selected randomly from an inventory of such households that had been conducted within 6 months prior to each survey. Teams were instructed to invite any child under 5 years old who was available on the day of the survey and had slept in the household the night before for participation. The resulting sample (average 24 children per community) covered approximately 20% of the under-five population in each survey, based on the 2010 census data and community lists developed by the GHS [3]. Thin and thick blood films were prepared in the field and later read by two qualified microscopists at the Noguchi Memorial Institute for Medical Research laboratory in Accra to determine microscopic parasitaemia. In cases of discordant readings, a senior microscopist determined the final result. Prior to analysis, children aged under 6 months were removed from the dataset, based on previous studies suggesting that young infants have enhanced malaria protection due to maternal antibodies [24, 25].

Using a hand-held Etrex© GPS device (Garmin), field technicians obtained GPS coordinates for a readily identifiable central point in each community, such as a church, school, or chief’s palace. GPS coordinates were not obtained at the household level because of feasibility and ethical considerations. The geo-coding of communities permitted the dataset to be enhanced with remotely sensed and geographic variables.

### Variables employed to model malaria parasite prevalence

Remote sensed variables Malaria transmission has been shown to be strongly related to satellite-derived environmental and socio-demographic factors [26]. Weiss et al. [27] carried out a comprehensive assessment of spatially gridded covariates that are likely to be associated with the malaria transmission cycle. Based on their results a suite of satellite and geographically derived covariates were assembled for modelling purposes (Table 1). The selection of environmental covariates was partly based on availability of raster data that closely matched the survey times. Elevation is widely used in malaria mapping due to its association with precipitation and temperature [27] and it was extracted from the 90-m resolution Shuttle Radar Topography Mission Digital Elevation Model (SRTM-DEM). The normalized difference vegetation index (NDVI), a proxy for vegetation cover, was obtained from Moderate Resolution Imaging Spectroradiometer (MODIS) products using the 16-day composite. Based on this product, the maximum NDVI was calculated within a 32-day period prior to the start date of each survey. Vegetation cover is a useful proxy for characterizing vector habitat for *Anopheles* spp. commonly found across Africa [28]. The land surface temperature (LST) for day and night was also obtained from MODIS (MOD11) products using the 8-day composites, which were then used to calculate a monthly average LST 32 days prior to survey start date. Temperature has been widely accepted to be an important component in malaria transmission, largely based on its influence on mosquito survival, development, breeding, and biting rates [8, 29, 30], whereas precipitation is a proxy for available stagnant water puddles that are ideal habitat for mosquito larvae. Previous studies have shown that a 9 to 12-week time lag exists between rainfall and onset of malaria transmission [31]. Therefore, the variable of cumulative rainfall for a 3-month period 30 days prior to survey start date was created, based on the Climate Hazards Group InfraRed Precipitation with Station (CHIRPS) data while long-term precipitation patterns at finer resolution than CHIRPS (1 km) were extracted from WorldClim datasets [32]. Long-term precipitation was included because of its higher spatial resolution compared to cumulative rainfall. Night-time lights for 2011 were obtained from National Oceanic and Atmospheric Administration (NOAA) satellite products at 1-km resolution [33], being a useful proxy for poverty and infrastructure (i.e., electrified housing are more likely to be walled and be less suitable habitat for vector development) [34]. Because densely populated areas are often poor habitat for *Anopheles* to breed [35], Population density at 100-m resolution was extracted from WorldPop [36] and validated this product using the census data for the two main urban towns of Bunkpurugu and Nakpanduri. Finally, land use products by MODIS were expected to be an important factor [27] for vector habitat, however it was not included because there was little variation across the study district [37].

**Table 1:** List of spatial predictors, including their sources and spatial resolution (maps of spatial covariates can be found in Additional file 1)

Variable	Definition (units)	Spatial resolution	Source
Remote-sensed			
Elevation	Height above sea level (m)	90 m	CGIAR SRTM [49]
Normalized difference vegetation index (NDVI)	Index of vegetation conditions. Ranges from − 1 (no vegetation) to 1 (complete vegetated)	250 m	NASA (Terra) MOD13A3 and (Aqua) MYD13A3 datasets [50]
Land surface temperature (LST)—day and night time	Kelvin (converted to °C)	1 km	NASA (Terra) MOD11A2 and (Aqua) MYD11A2 datasets [50]
Rainfall (seasonal 3 months cum.)	Actual cumulative 3-months rainfall (mm)	5 km	CHIRPS [51]
Long-term precipitation 1970–2000 (seasonal 3 months cum.)	Long-term cumulative 3 months rainfall based on average monthly rainfall (mm) data from 1970–2000	1 km	WorldClim [52]
Night-time lights	Average visible band digital number values ranging 1–63	1 km	NOAA [33]
Population density	Number of people per 100 sq m	100 m	WorldPop [36]
GIS-derived			
Distance to urban centre	Distance from urban centre (km)	1 km	Based on field data
Distance to health facility	Distance from active health facilities during study (km)	1 km	Based on field data
Distance to roads	Distance from established road-network (km)	1 km	CIESIN [46]
Distance to water bodies	Distance from permanent water bodies (km)	1 km	ESRI [45]
Accessibility to cities	Travel time to cities to assess inequalities in accessibility (travel time)	1 km	MAP [44]
Slope	Percentage rise in elevation	90 m	Derived from elevation product

#### GIS derived variables

In relation to the geographically derived variables, Euclidean distances from roads, urban centres, permanent water bodies, and active health facilities were calculated using ArcGIS 10.3 [38]. A 5-km buffer was created around the study area to control for edge effects that might affect distance calculations. The inclusion of these distance variables was based on their biological importance to malaria transmission: urban centres are known to be associated with lower malaria prevalence due to their infrastructural development and access to resources such as medication [39]. Access to health facilities has been shown to be a key determinant in child mortality and primary usage of health clinics declines with increased travel time and distance [40]. Roads are a proxy for accessibility [41, 42] and water bodies are directly linked to mosquito breeding sites [42, 43]. Accessibility to cities, developed by the Malaria Atlas Project (MAP), was also included as a metric to account for distance to cities, transport infrastructure and distribution of resources [44]. A geo-coded shape file with urban centres was created based on field work records. Urban centres were defined as villages with more than 5000 people in the 2010 census, resulting in two urban centres in the study region: Nakpanduri and Bunkpurugu. Field workers used GPS to record locations of active health facilities in each survey period. Shape files for water bodies and major roads were obtained from ESRI online [45] and the Center for International Earth Science [46], respectively. Finally, slope was derived from the SRTM elevation raster. A list of covariates and their details can be found in Table 1.

#### Adjusting the model for child age

The only individual-level variable used for modelling was child’s age. Age is known to be strongly associated with individual-level parasitaemia and therefore was accounted for in the model to generate age-adjusted malaria prevalence predictions [47]. Diggle et al. explored a similar approach of including non-spatial covariates for malaria mapping such as age and bed-net use to explain the non-spatial variation in model-based geostatistical methods [48]. A detailed risk analysis involving other individual-level variables for this same dataset is provided elsewhere [4].

### Selecting a suitable set of variables for prediction

The use of too many covariates can lead to over-fitting of the model, as well as multicollinearity [53, 54]. To address this issue, a two-step procedure, commonly used in mapping exercises [9, 55], was used to select the best predictors from Table 1. First, correlation between covariates was calculated, and in cases of high correlation (i.e., $R$ > 0.7), a single representative covariate was selected. The choice of which covariate to retain was based on the strength of association with malaria prevalence, spatial resolution, and its relevance to malaria epidemiology [27]. Second, a bi-directional, step-wise, regression analysis was run on a full multivariate model using the remaining covariates. The covariates associated with the model with the lowest Akaike information criterion (AIC) were used for subsequent analysis.

### Bayesian geostatistical model

A Bayesian hierarchical geostatistical model was fitted independently to data from each survey. Let the malaria status $M_{ijt}$ for child $i$ in village location $j$ in survey *t* be a binary variable, equal to 1 for a positive microscopy result and 0 otherwise. Using a probit regression framework, assume that $M_{ijt} = 1$ if the latent variable $z_{ijt}$ is greater than 0 and $M_{ijt} = 0$ otherwise. The latent variable $z_{ijt}$ is modelled as:

$$ z_{ijt} \sim N\left( {\alpha_{jt} + \varvec{x}_{{\varvec{ijt}}}^{\varvec{T}}\varvec{\beta}_{t} ,1} \right) $$

where $\varvec{x}_{{\varvec{ijt}}}^{\varvec{T}}$ is the design vector containing the age of each child, $\varvec{\beta}_{t}$ contains the corresponding regression coefficients, and $\alpha_{jt}$ is the village-survey level random-effect. Assume that:

$$ \varvec{\alpha}_{t} \sim N\left( {\varvec{W}_{t}\varvec{\gamma}_{t} ,\sigma_{t}^{2} {\varvec{\Sigma}}\left( {\rho_{t} } \right)} \right) $$

where $\varvec{W}_{t}$ is the design matrix and $\varvec{\gamma}_{t}$ is a vector of the corresponding coefficients. Furthermore, ${\varvec{\Sigma}}$ is the spatial correlation matrix, where the correlation between villages $k$ and $l$ is given by the exponential parametric function $exp\left( { - \frac{{d_{kl} }}{{\rho_{t} }}} \right)$. In this expression, $d_{kl}$ is the Euclidean distance between villages *k* and *l* and $\rho_{t}$ is the correlation decay parameter.

Finally, the priors are given by:

$$ \varvec{\beta}_{t} \sim N\left( {0,\varvec{I}} \right) $$

$$ \rho_{t} ,\sigma_{t} \sim Unif\left( {0,100} \right) $$

$$ \varvec{\gamma}_{t} \sim \varvec{N}\left( {0,{\mathbf{\rm T}}} \right) $$

where ${\mathbf{\rm T}}$ is a diagonal matrix with diagonal elements equal to [10,1,…,1]. A customized Gibbs Sampler programmed in R [56] was used to fit this model separately for each survey.

### Model validation

The geospatial model performance was assessed based on its out-of-sample predictive ability using a tenfold cross-validation approach for each survey. Villages in the data were randomly divided into 10 sub-sets. The model was trained on 9 of the 10 sub-sets, and then used the estimated parameters to predict the expected prevalence for the remaining withheld sub-set. This was repeated using each testing sub-set. The out-of-sample predictive skill of the geospatial Bayesian model was compared to that of a standard generalized linear regression model (GLM), which is a commonly used statistical framework for modelling this type of response variable. A temporal validation was also done for the Bayesian model, where the model was trained on one survey and used to predict prevalence for future surveys. These temporal predictions were stratified by season (i.e., rainy season surveys were used to predict future rainy season surveys but not dry season surveys). Two statistical metrics were used for all validations: the log-likelihood and the mean absolute error (MAE) in relation to malaria prevalence in each village.

### Extrapolation to neighbouring district

Once the geospatial model was fitted, the results were extrapolated to the neighbouring district: East Mamprusi. This district was chosen because it has similar topography and environment to Bunkpurugu–Yunyoo. Furthermore, a list of health facilities and urban centres for East Mamprusi was obtained from the GHS [57] and corroborated using expert information and the 2010 population census, respectively [22], enabling the determination of the distance to health facilities and urban centres. For an independent evaluation of the accuracy of these spatial extrapolation, the MAE was calculated between the predicted malaria prevalence rates in the 2011 rainy season and the 2011 Multiple Indicator Cluster Survey (MICS) prevalence estimates for locations that fell within this district [58].

## Results

### Malaria prevalence

The study included 10,518 children aged 6–59 months of age with a complete microscopy diagnostic test result for malaria parasitaemia collected in 438 communities across 3 rainy seasons and 3 dry seasons. The average age of participants was 31 months. The average community level prevalence was 44.46% (95% CI 43.83–45.74) during the entire study but prevalence varied considerably by survey, with a strong seasonal effect (Table 2).

**Table 2:** Average prevalence across each survey

Survey year	2010	2011		2012		2013
Season	Rainy	Dry	Rainy	Dry	Rainy	Dry
Number of children	1547	1762	1794	1804	1803	1808
Mean prevalence (%)	60	39	56	36	51	28
95% CI	57–62	36–41	54–58	34–38	49–54	25–29

### Variable selection

As mentioned in Methods, an initial set of 14 spatial covariates were chosen based on the malaria mapping literature [27] (Table 1). NDVI, rainfall, long-term precipitation, and daytime LST were highly correlated (R > 0.7). Because NDVI had the strongest association with microscopy and this covariate had the finest spatial resolution, NDVI was retained and the other three highly correlated covariates were excluded from the step-wise selection. Because accessibility was highly correlated with distance to health facility (R = 0.71), given that distance to health facility was based on ground-truth locations of active health facilities at the time of each survey, it was retained instead of accessibility. A step-wise, model-selection approach using AIC revealed that the most important covariates were distance to urban centre, distance to health facility, elevation, NDVI, distance to road, distance to water, LST at night, and night-time lights. These parameters were used for geostatistical predictions (parameter estimates can be found in Additional file 2).

### Geospatial model results

**Fig. 2:**
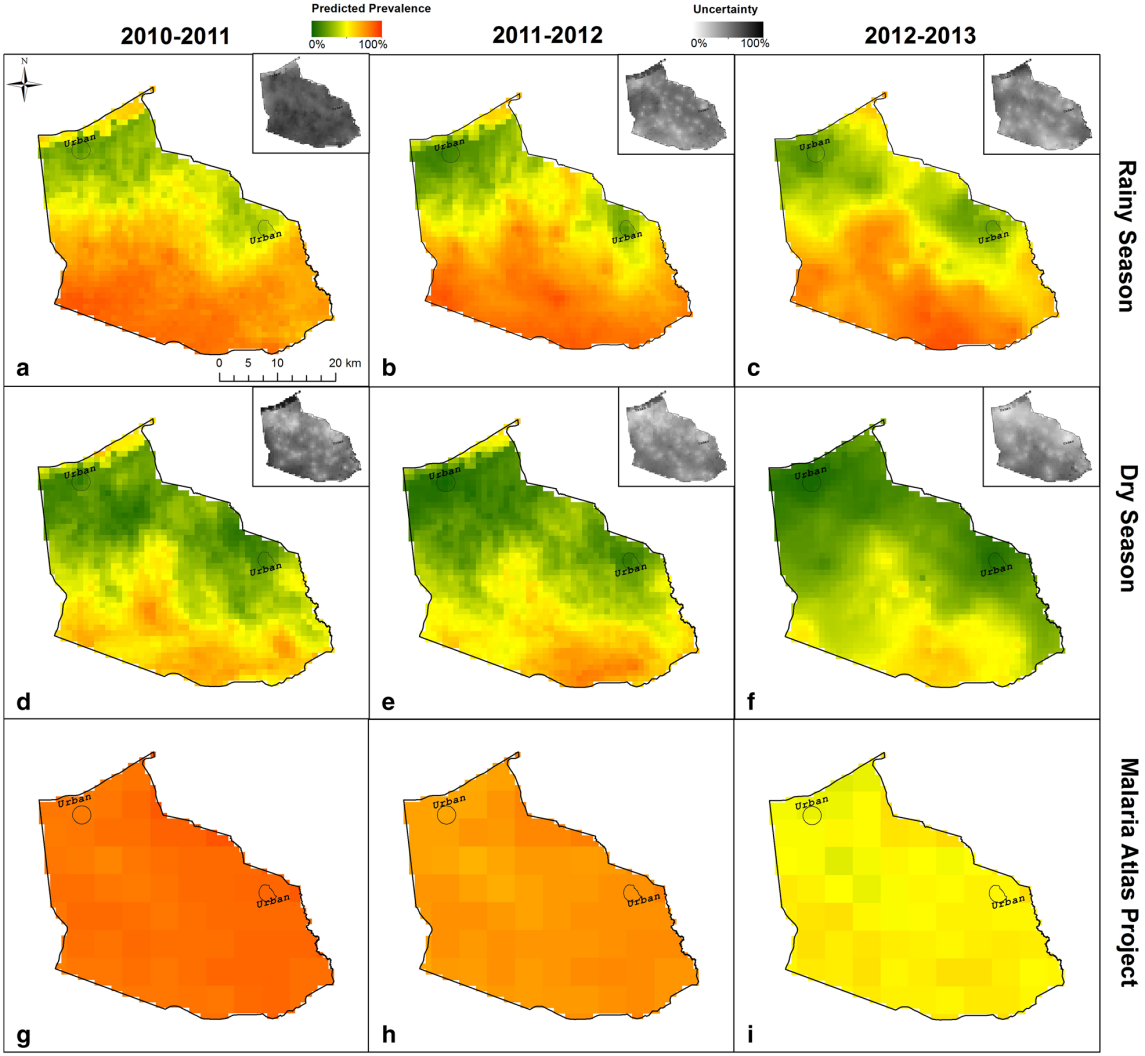
Strong seasonal and fine-scale spatial variability in age-adjusted predicted malaria prevalence. Top and middle panels (**a**–**f**) display the age-adjusted predicted mean prevalence for the rainy and dry seasons, respectively. Age-adjusted malaria prevalence is based on children with average age (i.e., 31 months). The bottom panels (**g**–**i**) display the MAP parasite prevalence surface for *P. falciparum* between ages 2 and 10 years [11]. Insets show the uncertainty in prevalence predictions (top right in each panel), given by the width of the 95% CI. High uncertainty is represented by darker grey

The Bayesian geostatistical model revealed a strong rural–urban gradient with prevalence generally increasing northeast to southwest (top and middle panels in Fig. 2) ranging from 19 to 90%. These results were particularly surprising given that the malaria prevalence maps for Ghana developed by the MAP suggest a much smaller range in prevalence (68–83%), without a clear geographical trend (bottom panels in Fig. 2). Malaria remained low in and around the urban centres throughout the study but this pattern was more distinct during rainy seasons versus dry seasons. The only notable temporal trend was a small reduction in prevalence in the southern portion of BYD in the final dry season.

The regression results indicate that increasing distance to urban centre was significantly associated with high malaria prevalence estimates during rainy seasons. Furthermore, higher elevation was linked to reduced parasite prevalence of malaria during the rainy seasons in 2010 and 2011, and the dry season in 2012. Finally, living further away from a health facility tended to increase the risk of high malaria prevalence, but this effect was only significant for dry season 2013. All other covariates showed no statistically significant association. Posterior estimates of the parameters used for predictive modelling are given in Additional file 2.

### Model validation

**Fig. 3:**
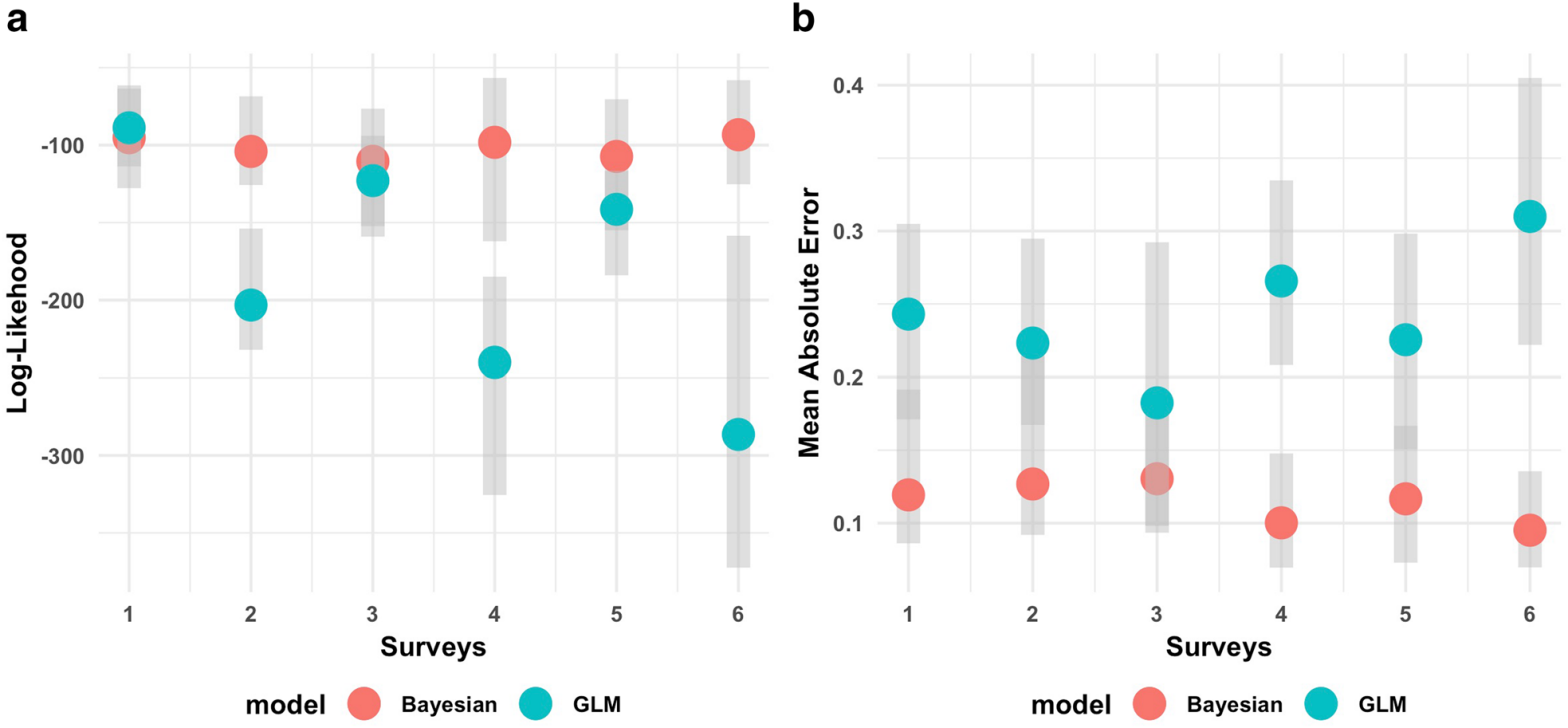
Model validation comparisons between Bayesian geospatial model and generalized linear model (GLM). Predictions based on the Bayesian geospatial model generally outperform the GLM. Two out-of-sample metrics were calculated; the log-likelihood for each survey (**a**) and MAE (**b**). Grey vertical polygons depict the range of outcomes based on the 10 cross-validation folds. Higher values for the log-likelihood and lower values for the MAE indicate better out-of-sample predictive skill

The model validation results using the average log-likelihood across all surveys (Fig. 3a) revealed that the Bayesian model generally had better out-of-sample predictive performance when compared to the GLM model, particularly during the dry seasons. Similar results also arose in relation to the mean absolute errors (Fig. 3b).

The temporal validation results reveal an average MAE of approximately 12.7%, without much difference in error for temporal predictions of 1 versus 2 years (Table 3). These MAE values are similar to those obtained when performing the tenfold cross-validation, suggesting that temporal predictions within a 1- or 2-year time interval are as accurate as spatial predictions.

**Table 3:** Temporal validation for surveys with temporal prediction lag of one and two years

Season	Year of estimation	Year of prediction	Mean absolute error
Rainy	2010	2011	0.124
Rainy	2011	2012	0.126
Rainy	2010	2012	0.132
Dry	2011	2012	0.126
Dry	2012	2013	0.124
Dry	2011	2013	0.126

### Extrapolation to neighbouring district

**Fig. 4:**
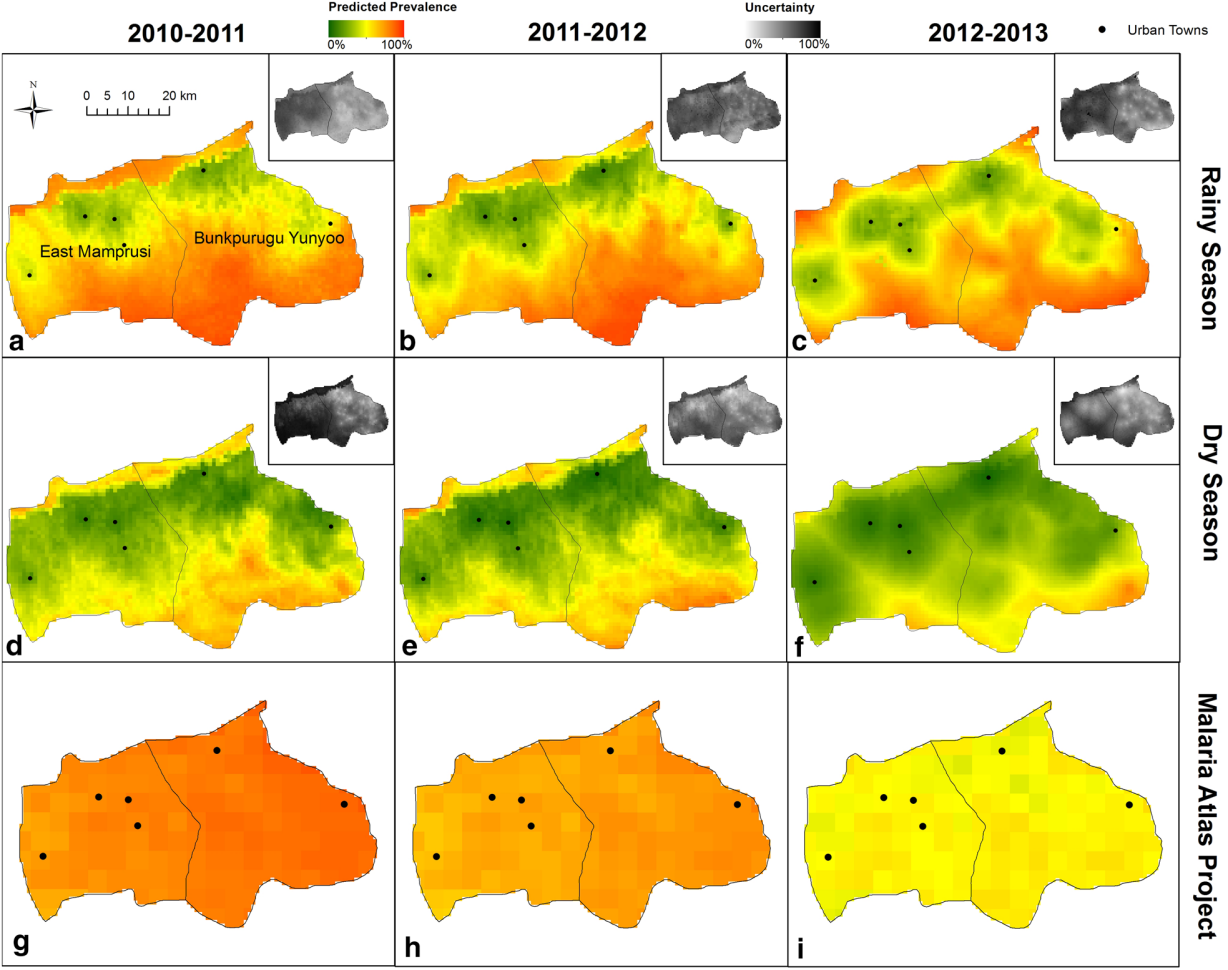
Extrapolated predicted prevalence for children in East Mamprusi and Bunkpurugu Yunyoo district. All six surveys including their corresponding uncertainty measures are displayed. Top and middle panels (**a**–**f**) display the age-adjusted predicted mean prevalence for the rainy and dry seasons, respectively. Age-adjusted malaria prevalence is based on children with average age (i.e., 31 months). The bottom panel (**g**–**i**) displays the MAP parasite prevalence surface for *P. falciparum* between ages 2 and 10 years [11]. The black dots represent urban centres with population greater than 5000 people. Insets show the uncertainty in prevalence predictions (top right in each panel), given by the width of the 95% CI. High uncertainty is represented by darker grey

Predicted malaria prevalence in East Mamprusi was substantially higher during the rainy season when compared to the dry season (Fig. 4). As expected, there is a distinct trend of lower transmission close to urban areas and the 2012–2013 dry seasons showed a significantly lower predicted prevalence. Finally, as expected, there is distinctly higher uncertainty across East Mamprusi compared to Bunkpurugu-Yunyoo for all surveys. The results for survey three (rainy season 2011) and MICS 2011 survey data were used to determine the reliability of these spatial extrapolations. MICS 2011 contained 10 clusters that fell in Bunkpurugu-Yunyoo and East Mamprusi district. Of these, six were in Bunkpurugu-Yunyoo and four in East Mamprusi. No location in survey data matched the MICS locations; so unfortunately, direct ground-truthing was not possible. The MAE was equal to 19.5 and 11.2% for Bunkpurugu-Yunyoo and East Mamprusi, respectively. The overall MAE for all 10 locations in both districts was 16%. While this may seem large, it is important to note that this margin of error is not substantially larger than those obtained with the tenfold cross-validation and temporal prediction exercises in Bunkpurugu-Yunyoo. Furthermore, this margin of error is enough to adequately distinguish the main spatial prevalence trends in the region, something that current national prevalence maps are not able to detect. For comparison purposes, the MEA for these 10 locations with the MAP 2011 estimates was 37.6% (graphical representation can be found in Additional file 2: Figure S1).

## Discussion

This study presents a Bayesian geostatistical analysis of the active surveillance data collected over a period covering high (rainy season) and low (dry season) transmission seasons in a region that has previously been characterized as uniformly high transmission. The application of high-resolution remote sensing data and geostatistics to develop malaria prevalence maps at a fine spatial resolution has revealed striking heterogeneity of malaria prevalence over a small geographic area, which were previously not expressed in country level and global mapping efforts [5, 11, 13]. For example, the malaria prevalence map for Ghana developed by the MAP for all survey years show predicted prevalence in BYD ranging from 68 to 83%, failing to encompass the range predicted by the model (19 to 90%) and to uncover the strong northeast to southwest trend that was detected. These differences can be explained in multiple ways. For instance, the prevalence surface from MAP is based on children 2–10 years old, focuses solely on *P. falciparum*, and relies on satellite imagery that was aggregated to 5 km and averaged temporally to suit their dataset’s temporal scale. In contrast, the results in this paper rely on satellite imagery that matches the dates of the surveys, and is based on children between 6 months and 5 years of age. Comparisons of maps based on different age ranges may lead to spurious conclusions, therefore a sensitivity analysis was conducted by reconstructing the prevalence data using the MAP age-adjustment model [59] to attain prevalence estimates for age 2 to 10 years and then re-created the prevalence maps per survey. A tenfold cross-validation estimates MAE being 36.9% and 12.5% on average across all surveys for MAP and the Bayesian model, respectively (Additional file 3). These results suggest that the main reason for these strikingly different prevalence maps is because the model took advantage of fine-scale malaria surveillance data, whereas MAP had to rely on spatially sparse data from national surveys (i.e., Demographic Health Surveys (DHS), MICS, MIS), in which the spatial coordinates are displaced slightly. Ultimately, these findings highlight the importance of fine-scale data to enable strategic allocation of resources and malaria prevention and control interventions.

The resulting maps predicted elevated risk during the rainy season, particularly in low-lying areas near the riverine plains. The prevalence across rainy seasons showed very little change implying that temporal change between years is limited, despite substantial within-year seasonal differences. Importantly, regardless of season and year, malaria prevalence was higher in the southern regions of the study area. Furthermore, the temporal validation also finds that 2-year ahead predictions of the spatial distribution of malaria prevalence were relatively accurate. Taken together, these results suggest that, in the absence of large-scale changes in malaria interventions, active data collection to spatially guide malaria prevention and control interventions might not be required multiple times within a year nor every single year, an important consideration given the high costs associated with data collection.

The maps in Fig. 2 also reveal a strong urban–rural relationship, where the urban areas experience lower rates of malaria prevalence when compared to the rural areas across all seasons and years. Although the association between lower malaria prevalence and urban areas has long been acknowledged [60, 61], it was found that even relatively modest urban centres (e.g., population 6000–12,000) in rural districts have a strong protective effect. This could be because these centres have fewer mosquito breeding sites [62], better housing conditions that provide protection against mosquito-human contact [63], and/or urban residents are more likely to have better access to medications [64]. A detailed analyses on the non-linear relationship between distance to urban centre and malaria prevalence in BYD has been reported elsewhere [4]. Acknowledging this strong effect of modest urban centres may help malaria prevention and control programmes better allocate their interventions. For example, after presenting the results to the local IRS programme, it was suggested that IRS could be prioritized to peri-urban and rural areas because urban areas, while easier to access, are more difficult to spray as heads of households are less likely to be at home during the day, making it difficult to reach the targeted structures to be sprayed.

Several environmental factors previously identified as driving malaria transmission in Ghana at the national level (e.g., rainfall and temperature [5, 64]) were not strong determinants of fine-scale malaria prevalence heterogeneity and were removed prior to modelling efforts. Furthermore, when fitting the geospatial model, many commonly used covariates were not statistically significant, including NDVI, distance to roads and water bodies, LST for night, and night-time lights. Although these covariates are identified as significant in the multivariate model used in step-wise selection, the effect of these covariates was probably well captured by the spatial random effects in the Bayesian model. Alternative reasons for these environmental covariates not being significant might be because, at small spatial scales, there is little variation in them and perhaps part of the effect of these variables is already being captured by distance to urban centre. It is possible that socio-economic variables (e.g., wealth) play a much more important role at this spatial scale than these environmental covariates. Finally, individual level non-spatial covariate were restricted to age only to avoid interpretation issues regarding the predicted prevalence surface. However, the model is designed such that more individual level covariates can be included. A more detailed study on the inference of socio-economic, demographic, environmental, and intervention at individual level has been conducted elsewhere [4].

Given the prohibitive cost associated with collecting fine-scale data on malaria prevalence over a large geographical region, an important aim of this paper was to determine the reliability of extrapolating model predictions to neighbouring districts. Using readily available survey data from MICS 2011, 10 clusters were obtained (six in Bunkpurugu Yunyoo and four in East Mamprusi) with observed malaria prevalence for October 2011, which is a small validation set. Nevertheless, these independent data suggest that the extrapolations results for both East Mamprusi and Bunkpurugu Yunyoo were still relatively accurate for the Bayesian model compared to published maps (16 and 37.6%, respectively) and therefore can still be useful for programmatic malaria prevention and control activities. Note that the extrapolation to a neighbouring district with was done using a similar environmental conditions and for which GIS data on urban city localities and active health facilities was readily available. Additional work will be needed to determine how extrapolation quality is impacted when environmental conditions differ substantially between districts, and to determine how generalizable these findings are.

The data presented here were based on the first six surveys during which a pyrethroid-based insecticide was applied across the district. Although the direct effects of the IRS intervention have not been accounted for, it was expected that IRS would result in declines of malaria prevalence across the district. Interestingly, the model results did not find much change over time with the exception of the sixth survey in the dry season of 2013. This suggests a potential lag in the impact of IRS across the district or the presence of substantial resistance to pyrethroids. Indeed, pyrethroids were subsequently switched to organophosphates and follow-on surveys not presented here revealed a significant drop in malaria prevalence [3]. Further work in this area would include modelling these follow-on surveys whilst adjusting for IRS intervention in the study area. The methodology developed in this paper would help identify how IRS spraying is impacting the spatial and temporal patterns of malaria in the region.

An important component of the model outputs are uncertainty estimates, defined here as the width of the 95% credible interval. The predicted uncertainty maps are indications of the precision around the mean estimated prevalence at a given location (i.e., at the pixel level), which helps users understand the robustness of the predicted mapped surface. Factors that contribute to uncertainty can include sparseness in the observed survey data (i.e., not enough observations in a given area), and/or inability of the model to explain the variability in the data [65, 66]. The results suggest that areas predicted to have elevated risk as well as higher uncertainty were mostly rural. These communities tend to be spread across larger geographic areas compared to the urban centres and the geographical coordinates, which were collected at easily recognizable landmarks in each community, may not be an accurate representation of the environment of a typical household in these rural communities. These results suggest that studies in many of these more rural areas may benefit from collection of GPS coordinates for each household or at least for clusters of households, instead of village-level coordinates, or sampling frameworks that are designed based on spatially stratified random sampling instead of solely on population proportional random sampling [67]. Finally, these uncertainty maps may help in the identification of areas in need of additional sampling [68].

## Conclusion

This study demonstrated how the use of high-resolution survey data and a geostatistical model can reveal local-scale spatial heterogeneity in an area previously assumed to be relatively uniform in terms of malaria risk. Characterizing the heterogeneity in the spatial distribution of malaria in this small geographic area enabled the identification of areas of high risk for which malaria prevention and control efforts can be strategically allocated to reduce malaria transmission in Bunkpurugu-Yunyoo, Ghana. Spatial extrapolations to neighbouring districts revealed that it is possible to take advantage of the rich data in one area to gain insight on the spatial heterogeneity in another, and temporal extrapolation results suggest that 2-year predictions can be made with similar accuracy as spatial predictions. These extrapolation results, together with limited seasonal and between-year variability in the location of hotspots, suggest that fine-scale data collection on malaria prevalence can be conducted less frequently, which is an important consideration for the long-term financial sustainability of these efforts, while still strategically guiding malaria prevention and control interventions.

## Additional files

**Additional file 1.** Spatial covariates maps for study region.

**Additional file 2.** Variable selection, posterior estimates and model comparisons.

**Additional file 3.** Sensitivity analysis to comparing age standardized local maps.

## Abbreviations

AIC: Akaike information criterion
BYD: Bunkpurugu-Yunyoo district
CHIRPS: Climate Hazards Group InfraRed Precipitation with Station data
CI: credible intervals
CIESIN: Center for International Earth Science Information Network
DHS: Demographic Health Surveys
GLM: Generalized linear regression model
GHS: Ghana Health Service
GIS: Geographical Information System
GPS: Geographic positioning system
MIS: Ghana Malaria Indicator Survey
IRS: indoor residual spraying
LLIN: long lasting insecticide treated nets
LST: land surface temperature
MAP: Malaria Atlas Project
MAE: mean absolute error
MODIS: Moderate Resolution Imaging Spectroradiometer
MICS: Multiple Indicator Cluster Survey
NASA: National Aeronautics and Space Administration
NMCP: National Malaria Control Programme
NOAA: National Oceanic and Atmospheric Administration
NDVI: normalized difference vegetation index
PMI: Presidents Malaria Initiative
SMC: seasonal malaria chemoprophylaxis
SRTM-DEM: Shuttle Radar Topography Mission Digital Elevation Model
