# Supplementary material for: Characterizing local-scale heterogeneity of malaria risk: a case study in Bunkpurugu-Yunyoo district in northern Ghana
Source: Malar J. 2019 Mar 15;18:81. doi: 10.1186/s12936-019-2703-4 (PMC6420752; doi:10.1186/s12936-019-2703-4)
Supplement: Supplementary file 1 — Additional file 1. Spatial covariates maps for study region. [file 12936_2019_2703_MOESM1_ESM.docx]

| 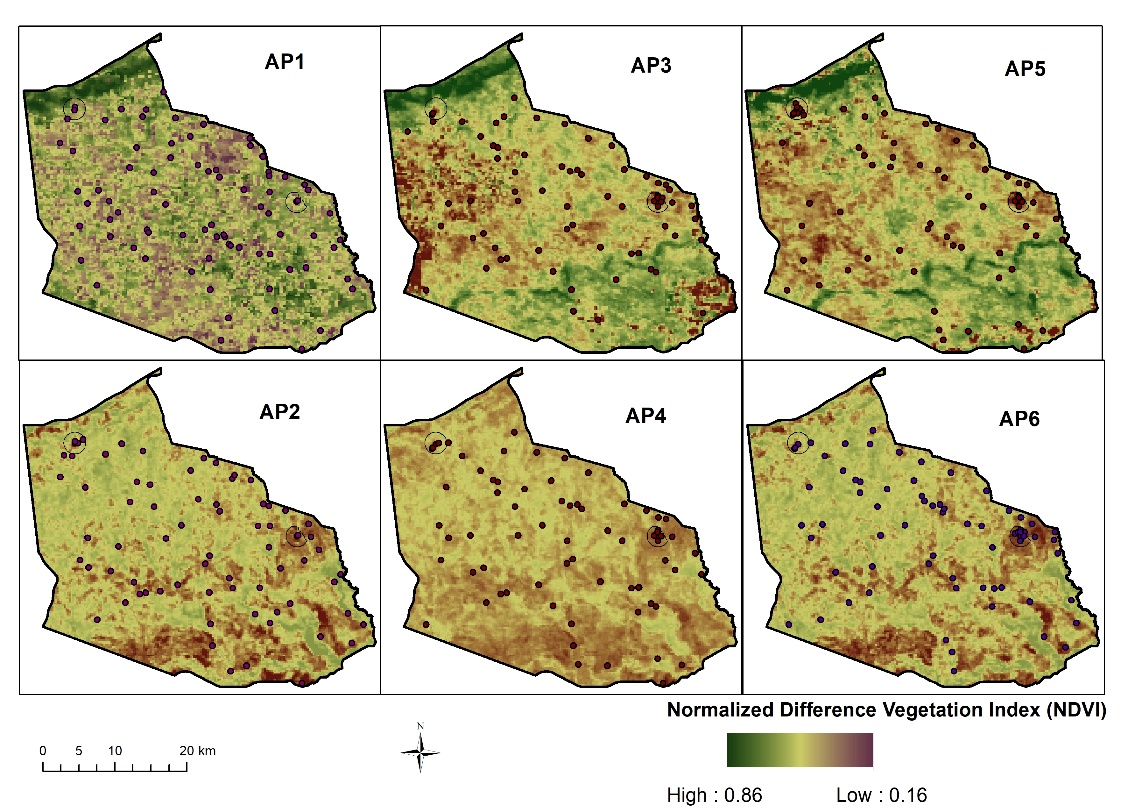 |
| --- |
| **Variable Name:** Vegetation (NDVI). **Seasonality:** 3 Rainy and 3 Dry seasons. **Type**: Environmental, Remote Sensed. |
|  |
| 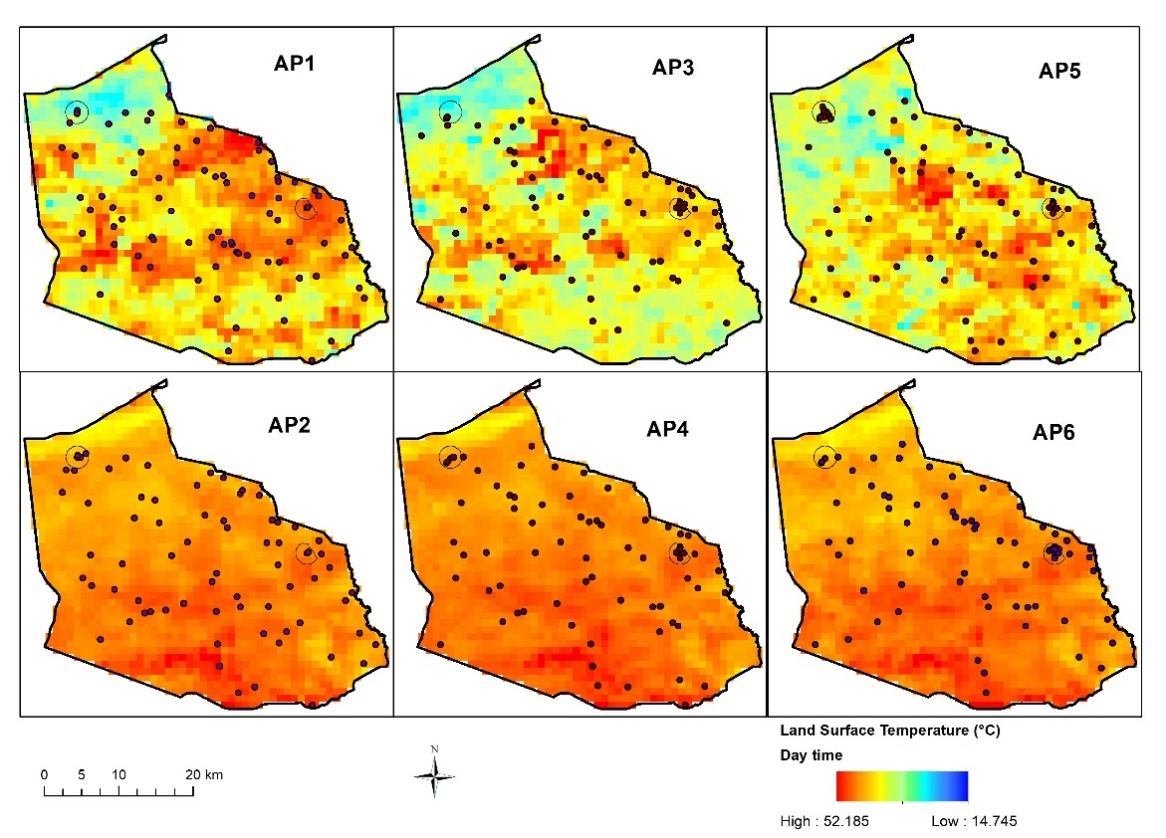 |
| **Variable Name:** Land surface temperature, Day. **Seasonality:** 3 Rainy and 3 Dry seasons. **Type**: Environmental, Remote Sensed. |
| 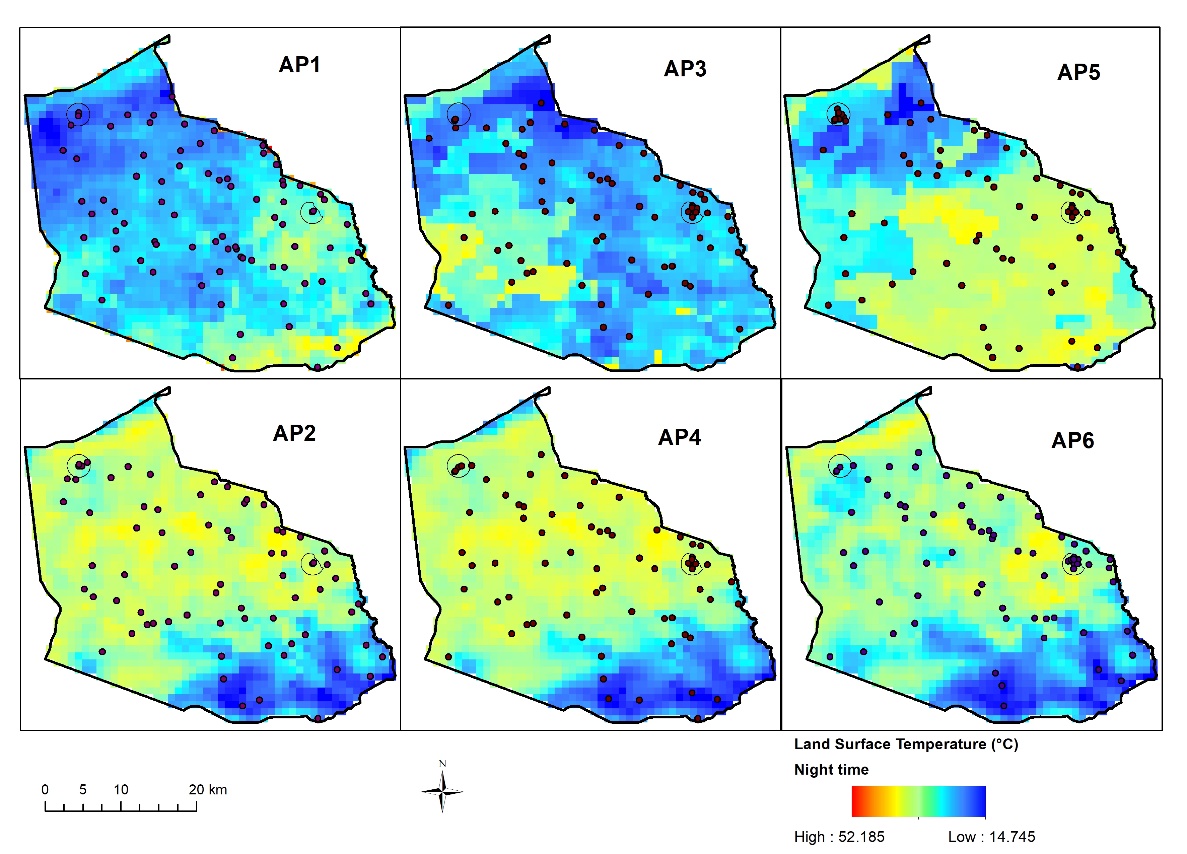 |
| **Variable Name:** Land surface temperature, night. **Seasonality:** 3 Rainy and 3 Dry seasons. **Type**: Environmental, Remote Sensed. |
|  |
| 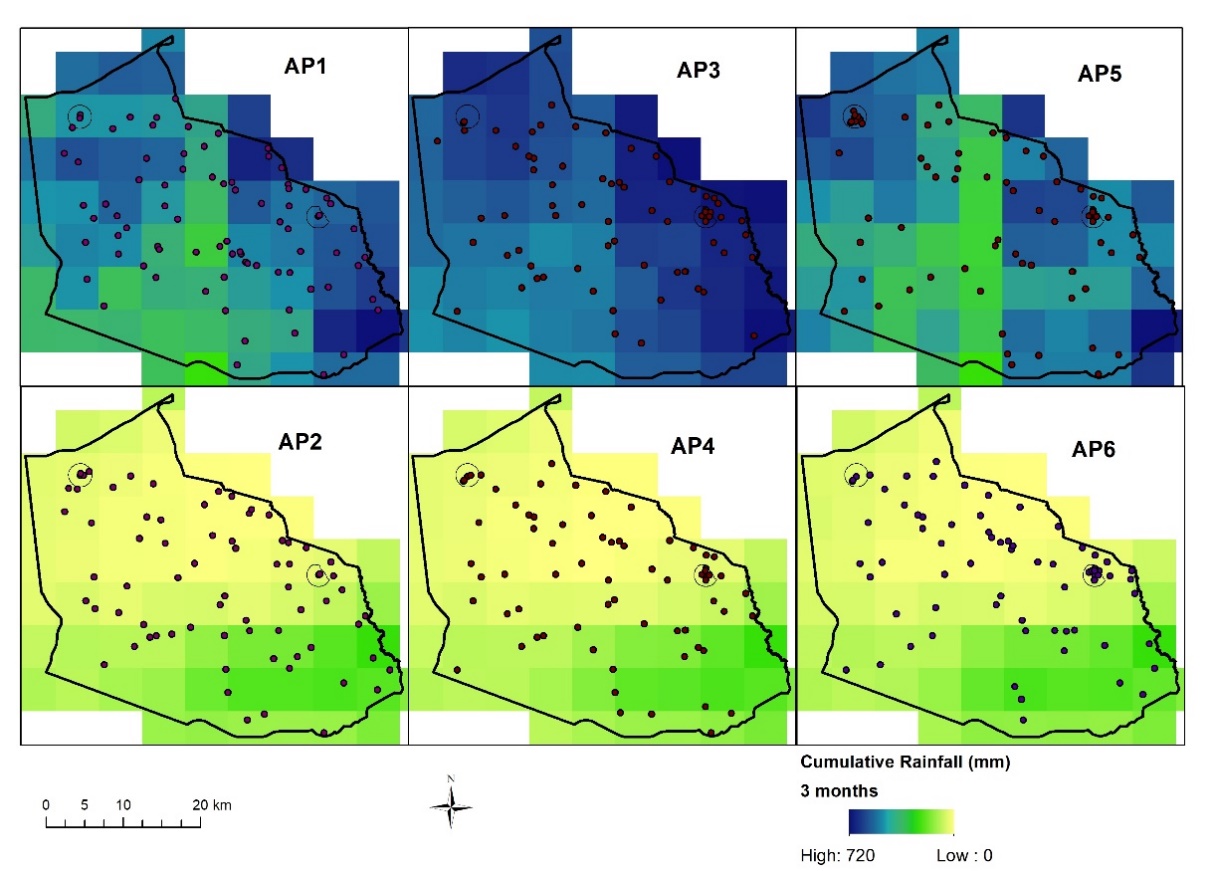 |
| **Variable Name:** Rainfall, 3-month cumulative. **Seasonality:** 3 Rainy and 3 Dry seasons. **Type**: Environmental, Remote Sensed. |
| 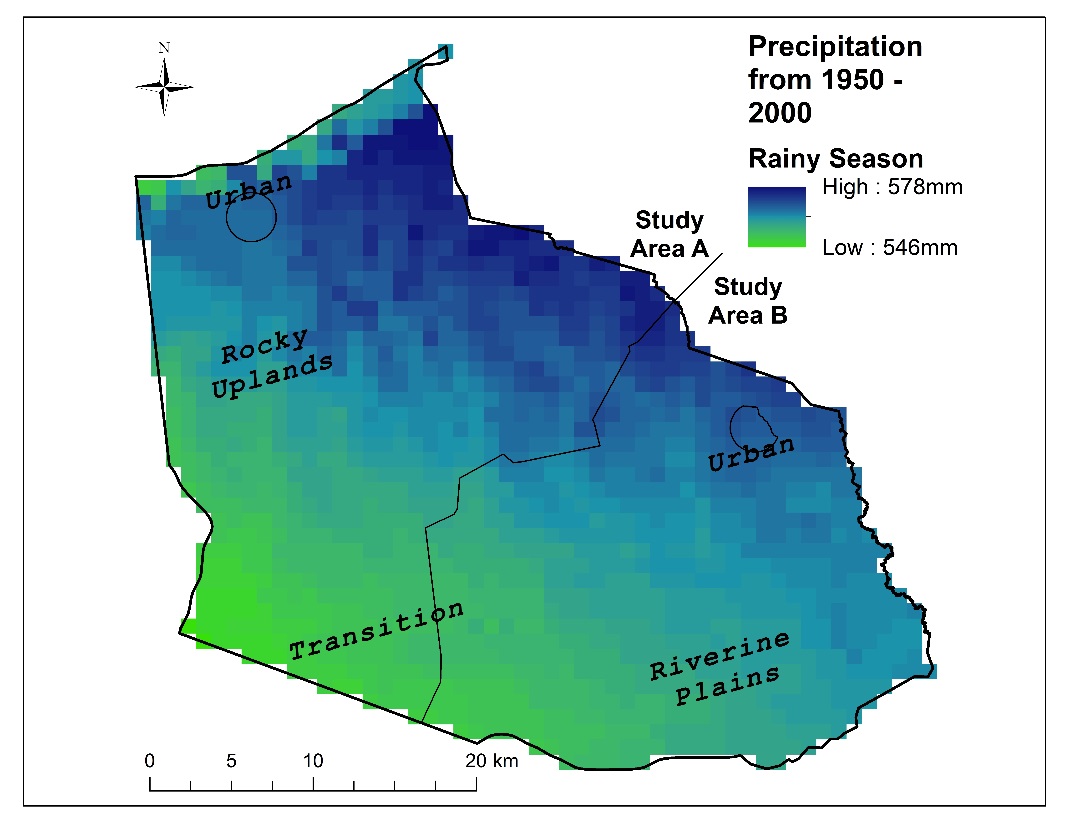 |
| **Variable Name:** Precipitation, 50-yr trend. **Seasonality:** Rainy. **Type**: Environmental, Remote Sensed. |
|  |
| 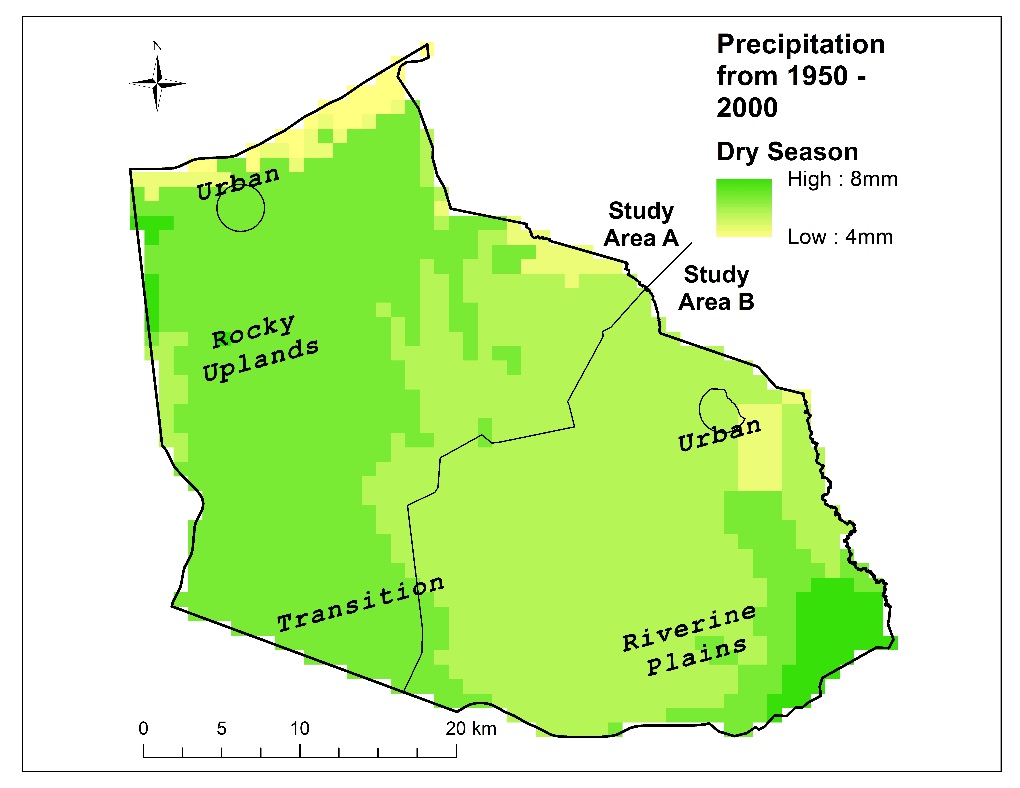 |
| **Variable Name:** Precipitation, 50-yr trend. **Seasonality:** Dry. **Type**: Environmental, Remote Sensed. |

| 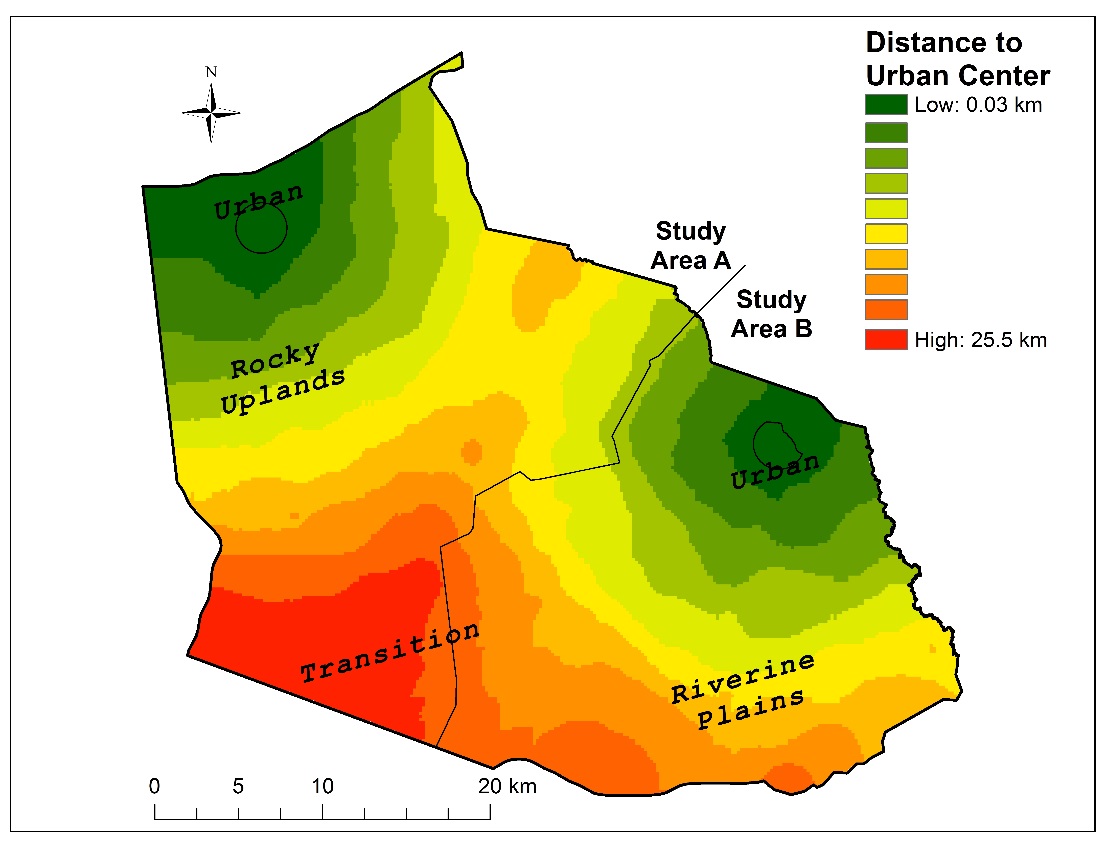 |
| --- |
| **Variable Name:** Distance to Urban Center. **Seasonality:** Fixed. **Type**: Anthropologic, GIS-Derived. |
|  |
| 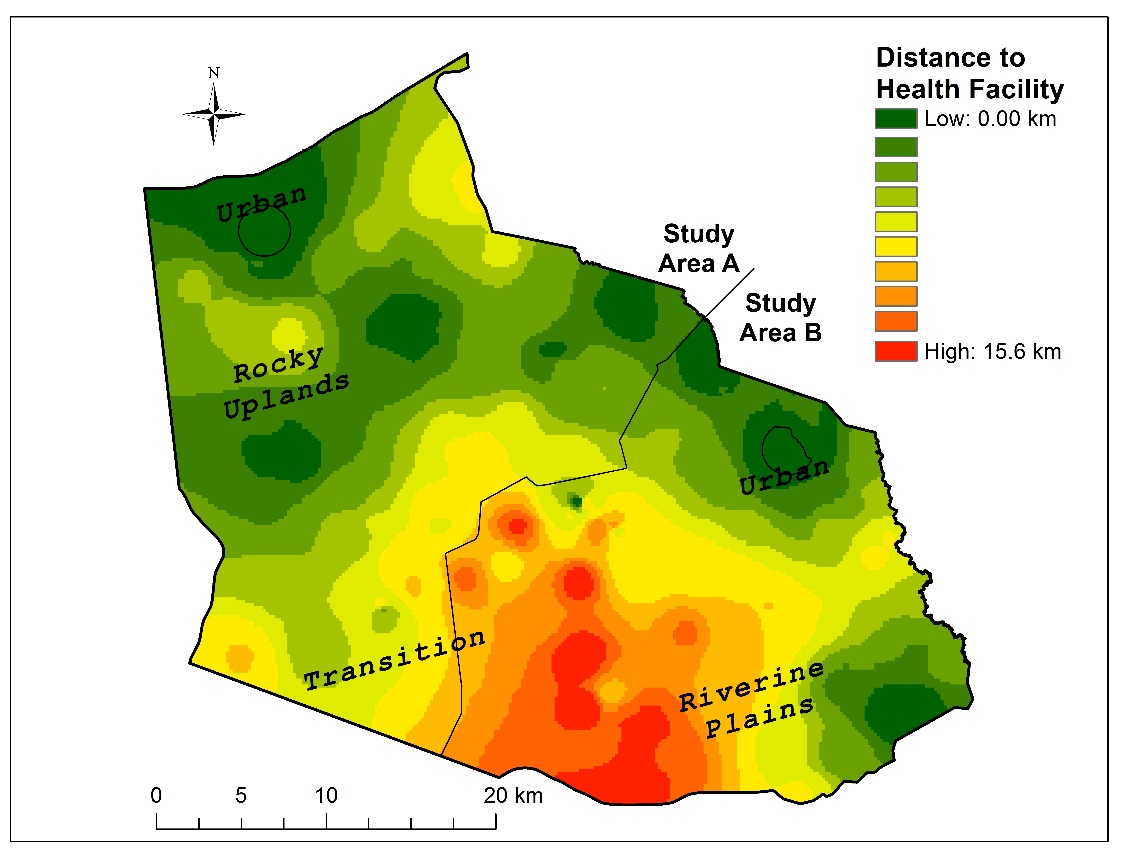 |
| **Variable Name:** Distance to Health Facility. **Seasonality:** Fixed. **Type**: Anthropologic, GIS Derived. |
| 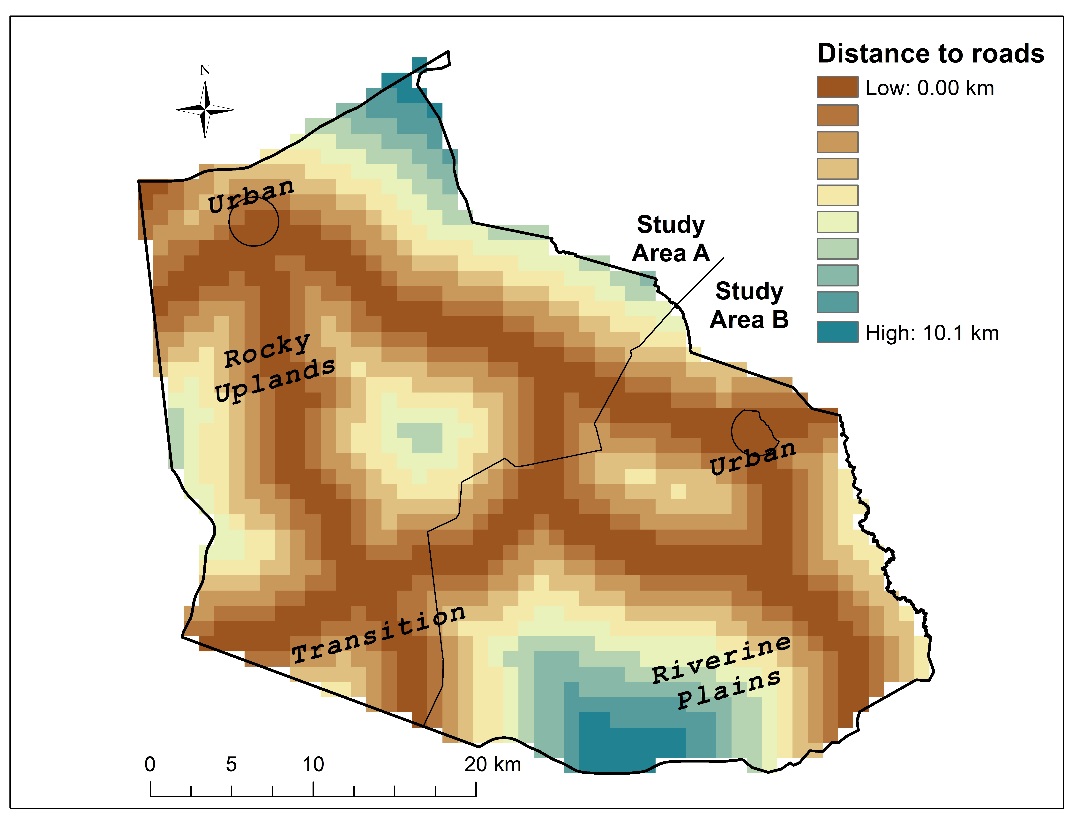 |
| **Variable Name:** Distance to Road. **Seasonality:** Fixed. **Type**: Environmental, GIS-Derived. |
|  |
| 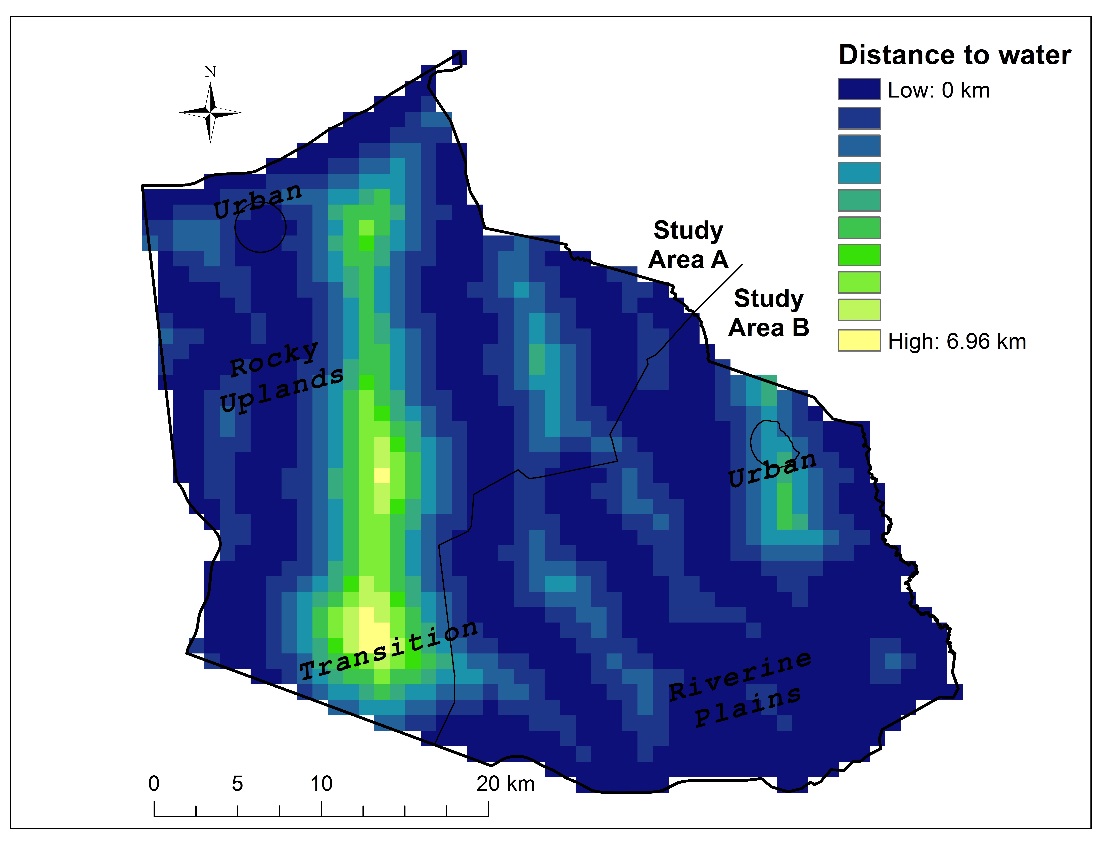 |
| **Variable Name:** Distance to Water Body. **Seasonality:** Fixed. **Type**: Environmental, GIS-Derived. |
| 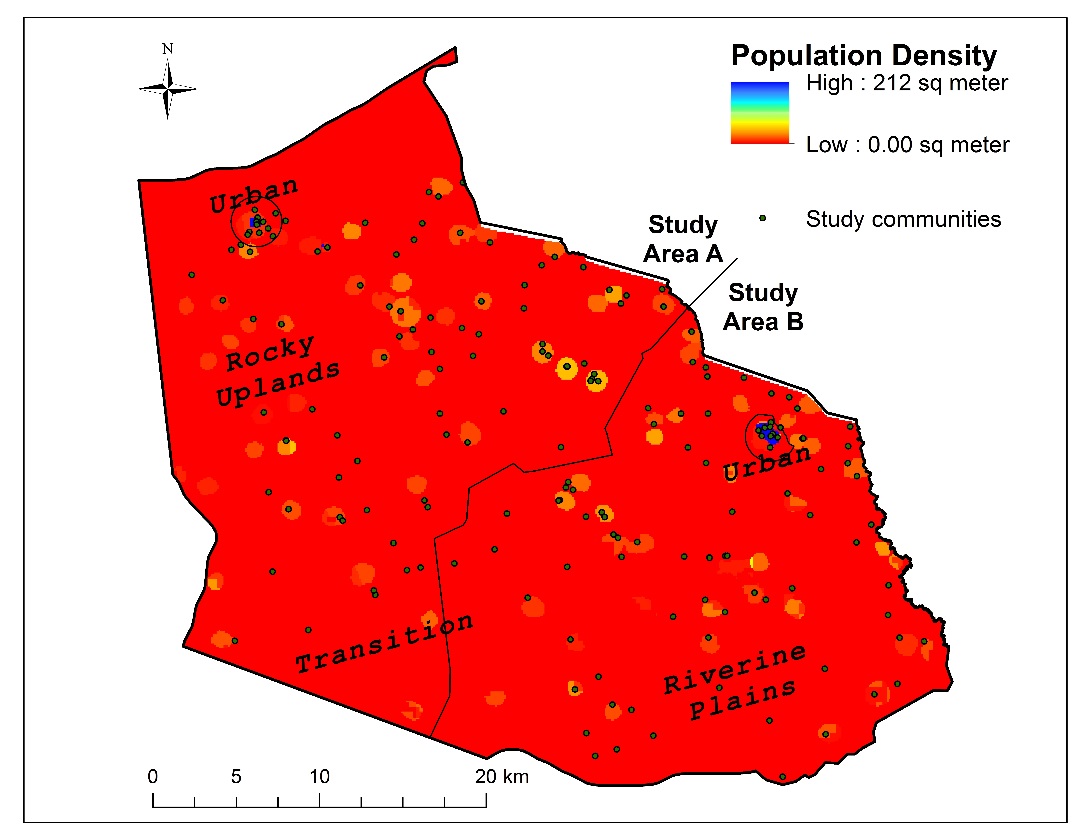 |
| **Variable Name:** Population Density (all ages). **Seasonality:** Fixed. **Type**: Anthropologic, GIS-Derived. |

| 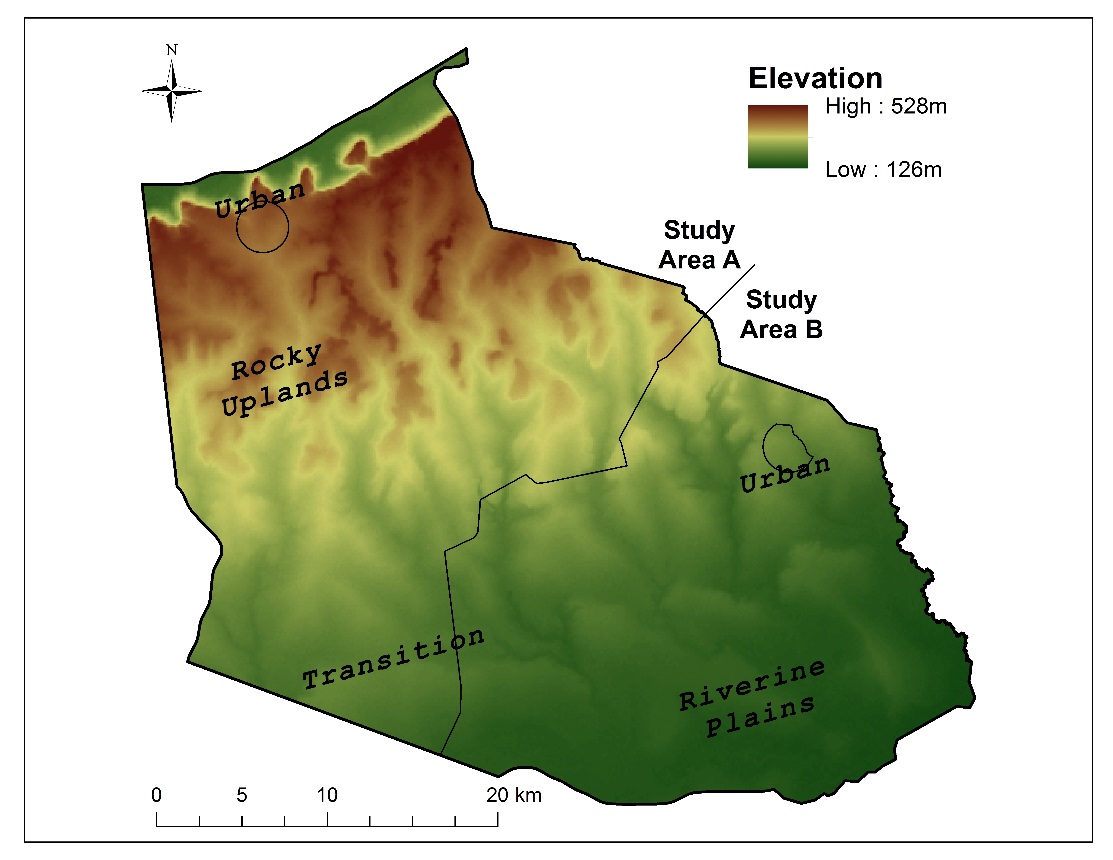 |
| --- |
| **Variable Name:** Elevation. **Seasonality:** Fixed. **Type**: Environmental, Remote Sensed. |
|  |
| 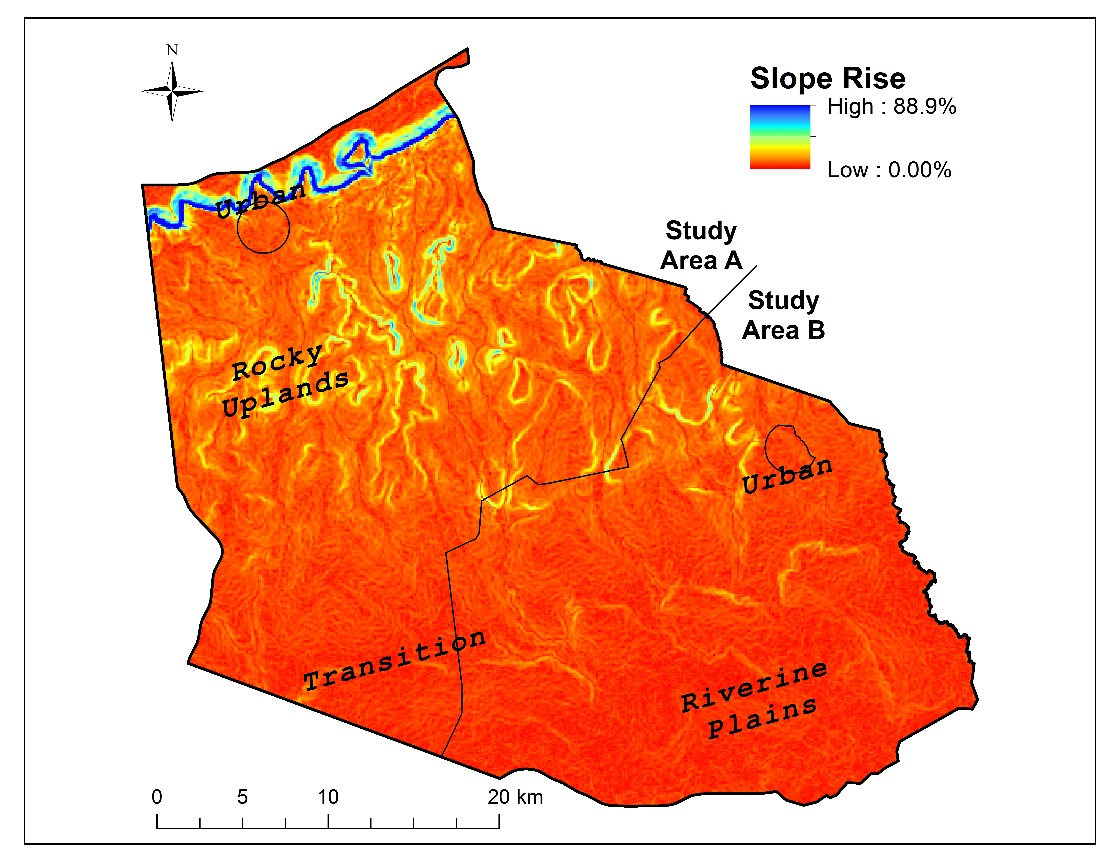 |
| **Variable Name:** Slope. **Seasonality:** Fixed. **Type**: Environmental, GIS-Derived. |
