# Supplementary material for: Characterizing local-scale heterogeneity of malaria risk: a case study in Bunkpurugu-Yunyoo district in northern Ghana
Source: Malar J. 2019 Mar 15;18:81. doi: 10.1186/s12936-019-2703-4 (PMC6420752; doi:10.1186/s12936-019-2703-4)
Supplement: Supplementary file 2 — Additional file 2. Variable selection, posterior estimates and model comparisons. [file 12936_2019_2703_MOESM2_ESM.docx]

**Additional file 2: Variable selection, posterior estimates and model comparisons.**

As part of the variable selection, alternative metrics based on travel times for access to health facilities were explored [1,2] but not included in this analysis, primarily because our derived covariates are made from ground-truth GPS based locations and we found that our list of active health facilities were more comprehensive than the available data. In regards to other distance metrics, we explored the use of travel time metrics readily available to describe accessibility [3] versus our own calculations based on Euclidean distance. We included both in our model, but found, not surprisingly, that travel time was highly correlated (R> 0.7) to distance to health facility and moderately correlated to distance to urban centres and roads (R > 0.6). We chose to retain Euclidean distance as our metric given it was a simpler metric to understand and compute. We also felt that in the case of our study area, which is rather small, remote and rural, travel time and distance are likely to provide similar surfaces. In the context of the district, the topography didn’t have large obstacles (e.g. mountains/lakes) and majority of the population do not own ground transportation so we felt it was safe to assume that the likeliest form of mobility was walking. We do believe our choice of Euclidean distance should be taken with some caution and may not particularly scale up to regional level, hence we feel it is important to explore, compare and evaluate the choice of unit to measure social behaviours such as accessibility. More information regarding all covariates used in our analysis are described in the main text in Table 1. Correlations for these covariates can be found in Table S1 and the posterior estimates for the final model after covariate selection can be found in Table S.2.

Table S1. Pearson correlation matrix of all covariates included in the model development. The darker orange represents correlations that are greater than 0.7 and yellow are those that are between 0.5 and 0.7. As part of our model only correlated variables greater than 0.7 were excluded.

|  | **Age** | **Distance to nearest HF** | **Distance to nearest UC** | **Elevation** | **Slope** | **Population density** | **NDVI** | **Cumulative Rainfall** | **Precipitation** | **LST (day)** | **LST (night)** | **Distance to nearest water body** | **Distance to nearest road** | **NTL** | **Access** |
| --- | --- | --- | --- | --- | --- | --- | --- | --- | --- | --- | --- | --- | --- | --- | --- |
| **Age** | 1.00 | -0.01 | -0.02 | 0.02 | 0.01 | 0.01 | 0.01 | 0.01 | 0.01 | 0.01 | -0.01 | 0.01 | 0.03 | -0.01 | 0.71 |
| **Distance to nearest HF** | -0.01 | 1.00 | 0.58 | -0.48 | -0.44 | -0.27 | 0.06 | 0.00 | 0.00 | 0.04 | -0.16 | -0.16 | 0.52 | -0.47 | 0.66 |
| **Distance to nearest UC** | -0.02 | 0.58 | 1.00 | -0.31 | -0.27 | -0.31 | 0.02 | -0.03 | -0.01 | 0.04 | -0.13 | 0.03 | 0.35 | -0.62 | -0.21 |
| **Elevation** | 0.02 | -0.48 | -0.31 | 1.00 | 0.65 | 0.15 | 0.01 | 0.02 | 0.03 | -0.09 | 0.07 | 0.11 | -0.18 | 0.10 | -0.28 |
| **Slope** | 0.01 | -0.44 | -0.27 | 0.65 | 1.00 | 0.02 | 0.02 | 0.01 | 0.01 | -0.06 | 0.14 | 0.10 | -0.16 | 0.00 | -0.04 |
| **Population density** | 0.01 | -0.27 | -0.31 | 0.15 | 0.02 | 1.00 | -0.12 | -0.04 | -0.04 | 0.04 | 0.00 | -0.01 | -0.15 | 0.45 | -0.22 |
| **NDVI** | 0.01 | 0.06 | 0.02 | 0.01 | 0.02 | -0.12 | 1.00 | 0.95 | 0.96 | -0.94 | 0.35 | 0.00 | 0.02 | -0.07 | 0.05 |
| **Cumulative Rainfall** | 0.01 | 0.00 | -0.03 | 0.02 | 0.01 | -0.04 | 0.95 | 1.00 | 0.99 | -0.96 | 0.40 | 0.03 | -0.02 | 0.02 | -0.01 |
| **Precipitation** | 0.01 | 0.00 | -0.01 | 0.03 | 0.01 | -0.04 | 0.96 | 0.99 | 1.00 | -0.98 | 0.37 | 0.03 | -0.01 | 0.01 | 0.01 |
| **LST (day)** | 0.01 | 0.04 | 0.04 | -0.09 | -0.06 | 0.04 | -0.94 | -0.96 | -0.98 | 1.00 | -0.31 | -0.01 | 0.01 | -0.01 | 0.01 |
| **LST (night)** | -0.01 | -0.16 | -0.13 | 0.07 | 0.14 | 0.00 | 0.35 | 0.40 | 0.37 | -0.31 | 1.00 | 0.21 | -0.28 | 0.06 | -0.20 |
| **Distance to nearest water body** | 0.01 | -0.16 | 0.03 | 0.11 | 0.10 | -0.01 | 0.00 | 0.03 | 0.03 | -0.01 | 0.21 | 1.00 | -0.23 | 0.20 | -0.16 |
| **Distance to nearest road** | 0.03 | 0.52 | 0.35 | -0.18 | -0.16 | -0.15 | 0.02 | -0.02 | -0.01 | 0.01 | -0.28 | -0.23 | 1.00 | -0.27 | 0.64 |
| **NTL** | -0.01 | -0.47 | -0.62 | 0.10 | 0.00 | 0.45 | -0.07 | 0.02 | 0.01 | -0.01 | 0.06 | 0.20 | -0.27 | 1.00 | -0.45 |
| **Access** | 0.71 | 0.66 | -0.21 | -0.28 | -0.04 | -0.22 | 0.05 | -0.01 | 0.01 | 0.01 | -0.20 | -0.16 | 0.64 | -0.45 | 1.00 |

Table S2. Posterior estimates of mean (95% Bayesian credible intervals) for model parameters

|  | Posterior Mean (95% BCI) | | | | | |
| --- | --- | --- | --- | --- | --- | --- |
| Year | **2010** | **2011** | **2011** | **2012** | **2012** | **2013** |
| Season | **Rainy** | **Dry** | **Rainy** | **Dry** | **Rainy** | **Dry** |
| Individual Covariates |  |  |  |  |  |  |
| $\boldsymbol{\beta}_{\boldsymbol{1}}$ - Age | **0.224***  **(0.154 - 0.291)** | **0.364***  **(0.297 - 0.435)** | **0.199(**  **(0.133 - 0.265)** | **0.428***  **(0.36 - 0.498)** | **0.243***  **(0.177 - 0.309)** | **0.353***  **(0.282 - 0.423)** |
| Community level covariates |  |  |  |  |  |  |
| $\boldsymbol{\gamma}_{\boldsymbol{0}}$ - Intercept | 0.267  (-0.348 - 1.015) | -0.488  (-1.549 - 0.61) | 0.055  (-0.166 - 0.27) | -0.635  (-1.097 - -0.303) | -0.066  (-0.718 - 0.637) | -0.952  (-1.884 - -0.289) |
| $\boldsymbol{\gamma}_{\boldsymbol{1}}$ - Distance to Health Facility | 0.115  (-0.051 - 0.292) | 0.027  (-0.237 - 0.281) | 0.043  (-0.19 - 0.274) | 0.019  (-0.196 - 0.238) | 0.223  (-0.019 - 0.494) | **0.21***  **(0.023 - 0.405)** |
| $\boldsymbol{\gamma}_{\boldsymbol{2}}$ - Distance to Roads | -0.017  (-0.152 - 0.124) | 0.041  (-0.159 - 0.259) | 0.025  (-0.143 - 0.191) | 0.122  (-0.039 - 0.292) | 0.166  (-0.017 - 0.353) | 0.008  (-0.158 - 0.174) |
| $\boldsymbol{\gamma}_{\boldsymbol{3}}$ - Distance to Urban Center | **0.229***  **(0.011 - 0.436)** | 0.23  (-0.19 - 0.558) | **0.413***  **(0.166 - 0.659)** | 0.227  (-0.042 - 0.468) | **0.361***  **(0.057 - 0.658)** | 0.259  (-0.029 - 0.541) |
| $\boldsymbol{\gamma}_{\boldsymbol{4}}$ - Distance to Water | 0.034  (-0.08 - 0.147) | -0.013  (-0.172 - 0.141) | -0.085  (-0.243 - 0.065) | 0.039  (-0.098 - 0.179) | 0.029  (-0.103 - 0.159) | -0.01  (-0.155 - 0.135) |
| $\boldsymbol{\gamma}_{\boldsymbol{5}}$ - Elevation | **-0.344***  **(-0.619 - -0.082)** | -0.35  (-0.948 - 0.03) | **-0.278***  **(-0.477 - -0.07)** | **-0.33***  **(-0.588 - -0.076)** | -0.085  (-0.407 - 0.317) | -0.092  (-0.372 - 0.248) |
| $\boldsymbol{\gamma}_{\boldsymbol{6}}$ - Land Surface Temperature (Night) | -0.058  (-0.209 - 0.098) | -0.107  (-0.364 - 0.147) | 0.009  (-0.162 - 0.178) | -0.111  (-0.34 - 0.092) | 0.047  (-0.128 - 0.232) | -0.025  (-0.192 - 0.157) |
| $\boldsymbol{\gamma}_{\boldsymbol{7}}$ - NDVI | 0.049  (-0.04 - 0.141) | 0.054  (-0.091 - 0.196) | -0.024  (-0.168 - 0.12) | 0.054  (-0.084 - 0.184) | 0.001  (-0.133 - 0.138) | -0.006  (-0.145 - 0.137) |
| $\boldsymbol{\gamma}_{\boldsymbol{8}}$ - Nighttime Lights | -0.037 (-0.179 - 0.103) | -0.073  (-0.269 - 0.118) | -0.063  (-0.296 - 0.162) | -0.119  (-0.359 - 0.106) | 0.091  (-0.145 - 0.327) | -0.029  (-0.282 - 0.227) |
| Spatial Parameters |  |  |  |  |  |  |
| $\boldsymbol{\sigma}^{\boldsymbol{2}}$ – spatial variance | **0.162**  **(0.021 - 0.891)** | **0.127**  **(0.02 - 0.773)** | **0.025**  **(0.016 - 0.057)** | **0.056**  **(0.017 - 0.319)** | **0.105**  **(0.017 - 0.709)** | **0.117**  **(0.017 - 0.656)** |
| $\boldsymbol{\rho}$ - correlation decay | **0.238**  **(0.04 - 1.079)** | **0.662**  **(0.127 - 3.669)** | **0.199**  **(0.082 - 0.399)** | **0.198**  **(0.049 - 0.802)** | **0.341**  **(0.071 - 1.796)** | **0.338**  **(0.062 - 1.763)** |

Figure S1. Mean absolute error comparison between Bayesian model, including East Mamprusi extrapolations, and Malaria Atlas Project (MAP) predictions using MICS 2011 data. The error is displayed for Bunkpurugu-Yunyoo (Blue), East Mamprusi District (Red) and both districts in total (Green).


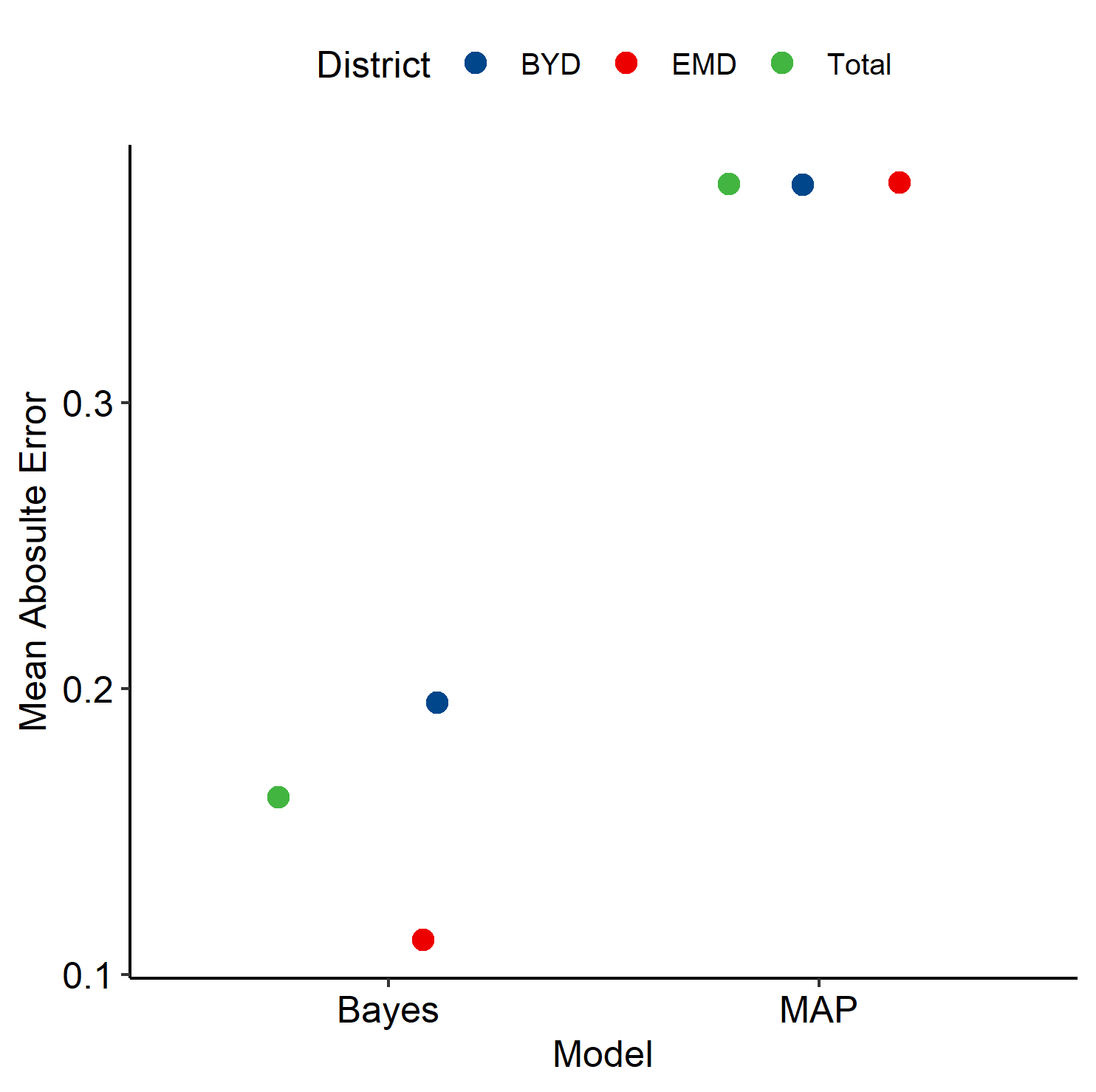


References

1. Ouma PO, Maina J, Thuranira PN, Macharia PM, Alegana VA, English M, et al. Access to emergency hospital care provided by the public sector in sub-Saharan Africa in 2015: a geocoded inventory and spatial analysis. Lancet Glob Heal. 2018;

2. Alegana VA, Maina J, Ouma PO, Macharia PM, Wright J, Atkinson PM, et al. National and sub-national variation in patterns of febrile case management in sub-Saharan Africa. Nat Commun. 2018;9:4994.

3. Weiss DJ, Nelson A, Gibson HS, Temperley W, Peedell S, Lieber A, et al. A global map of travel time to cities to assess inequalities in accessibility in 2015. Nature. 2018;
