# Supplementary material for: Characterizing local-scale heterogeneity of malaria risk: a case study in Bunkpurugu-Yunyoo district in northern Ghana
Source: Malar J. 2019 Mar 15;18:81. doi: 10.1186/s12936-019-2703-4 (PMC6420752; doi:10.1186/s12936-019-2703-4)
Supplement: Supplementary file 3 — Additional file 3. Sensitivity analysis to comparing age standardized local maps. [file 12936_2019_2703_MOESM3_ESM.docx]

**Additional file 3:** **Prediction comparison to Malaria Atlas Project results.**

As part of a sensitivity analysis we used the R package *malariaAtlas* and converted our current prevalence estimates from age 0.5 to 5 years to 2 to 10 years. We re-fit the model using R-INLA using a binomial model for prevalence. Covariates included in the modelling were distance to urban center, distance to health facility, elevation, NDVI, distance to road, distance to water, land surface temperature at night, and night-time lights. Predictions were made at a 1km spatial pixel resolution (figure S3.1). Please note that globally available MAP products are provided at 5km spatial resolution. We compared the appropriate MAP year to the appropriate survey. i.e., Survey 1 was done in October 2010 so MAP year 2010 was used for comparison. We ran a 10-fold cross validation on all locations to compare the mean absolute error for these maps against the malaria atlas project predictions at the same locations (figure S3.2).


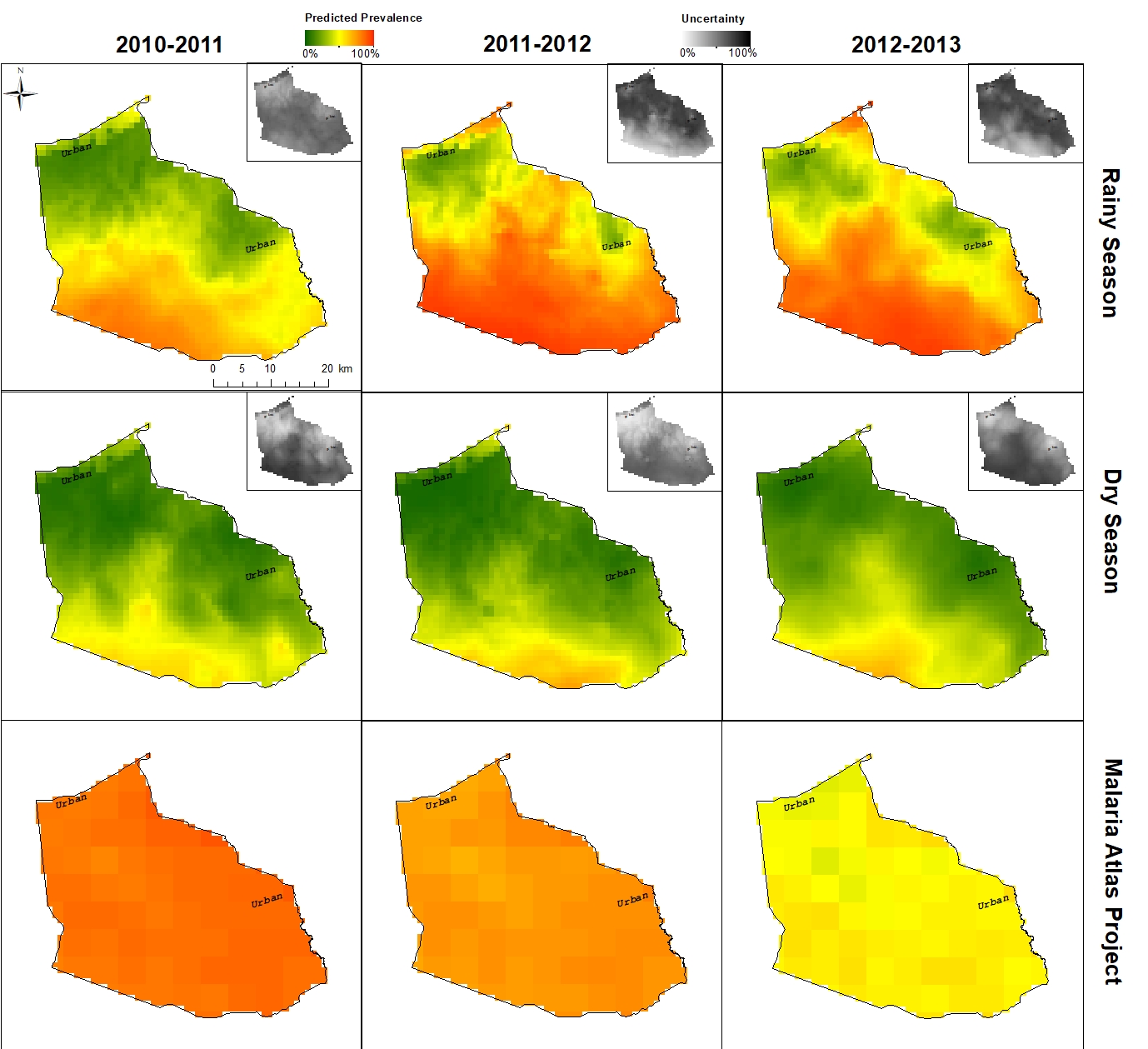


**Figure S3.1: Strong seasonal and fine-scale spatial variability in predicted malaria prevalence for ages 2 to 10 years**. Top and middle panels (A, B, C, D, E and F) display the age-adjusted predicted mean prevalence for the rainy and dry seasons, respectively. Age-adjusted malaria prevalence is based on children standardization techniques used by malaria atlas project. The bottom panels (G, H and I) display the Malaria Atlas Project parasite prevalence surface for Plasmodium falciparum between ages 2-10 years [8]. Insets show the uncertainty in prevalence predictions (top right in each panel), given by the width of the 95% credible intervals. High uncertainty is represented by darker grey.


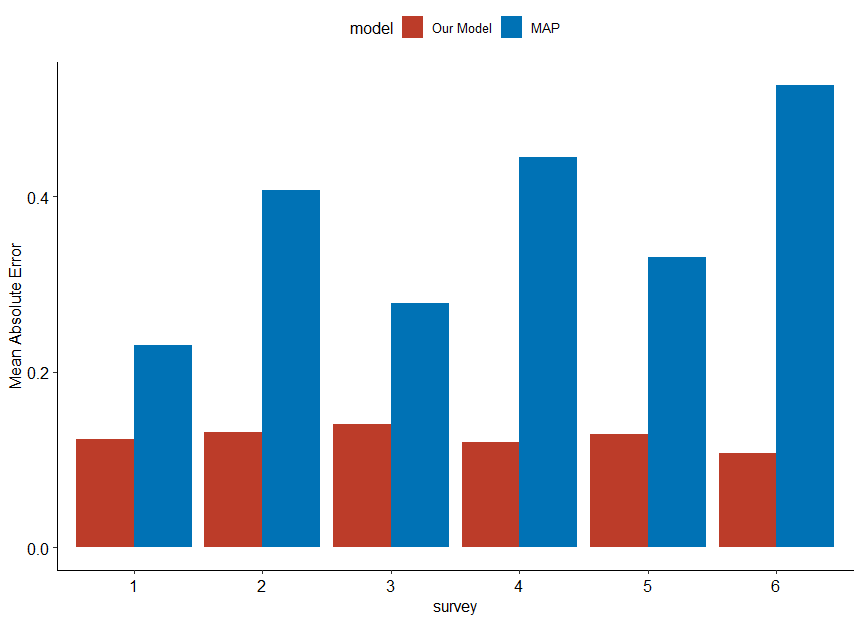


***Figure S3.2:*** **Model validation comparisons between our model and malaria atlas project.** We rely on the 10-fold cross-validation to calculate the mean absolute error. The blue bars are the error for prevalence estimates from MAP per survey and red represents our model error values. Higher values for the log-likelihood and lower values for the mean absolute error indicate better out-of-sample predictive skill.
